# A systematic review of meta-analyses assessing the validity of tumour response endpoints as surrogates for progression-free or overall survival in cancer

**DOI:** 10.1038/s41416-020-01050-w

**Published:** 2020-09-11

**Authors:** Katy Cooper, Paul Tappenden, Anna Cantrell, Kate Ennis

**Affiliations:** ScHARR, University of Sheffield, Sheffield, UK

**Keywords:** Cancer, Cancer

## Abstract

**Background:**

Tumour response endpoints, such as overall response rate (ORR) and complete response (CR), are increasingly used in cancer trials. However, the validity of response-based surrogates is unclear. This systematic review summarises meta-analyses assessing the association between response-based outcomes and overall survival (OS), progression-free survival (PFS) or time-to-progression (TTP).

**Methods:**

Five databases were searched to March 2019. Meta-analyses reporting correlation or regression between response-based outcomes and OS, PFS or TTP were summarised.

**Results:**

The systematic review included 63 studies across 20 cancer types, most commonly non-small cell lung cancer (NSCLC), colorectal cancer (CRC) and breast cancer. The strength of association between ORR or CR and either PFS or OS varied widely between and within studies, with no clear pattern by cancer type. The association between ORR and OS appeared weaker and more variable than that between ORR and PFS, both for associations between absolute endpoints and associations between treatment effects.

**Conclusions:**

This systematic review suggests that response-based endpoints, such as ORR and CR, may not be reliable surrogates for PFS or OS. Where it is necessary to use tumour response to predict treatment effects on survival outcomes, it is important to fully reflect all statistical uncertainty in the surrogate relationship.

## Background

Decisions about the use of new and existing health technologies should ideally be informed by estimates of treatment effects derived from high-quality randomised controlled trials (RCTs), which measure patient-relevant endpoints over a clinically appropriate timeframe. Such “final” endpoints typically involve the measurement of health benefits, which reflect aspects of the disease, and its treatment, which are important to patients (and potentially also their carers) and which relate to “how the patient feels, functions or survives”.^1^ In the context of advanced/metastatic cancer, the key matter of concern is often whether the use of a given health technology leads to improvements in overall survival (OS; a final endpoint) compared to existing standard treatments. However, the estimation of treatment effects on OS may be subject to numerous problems, including potential confounding resulting from the use of post-progression treatments, insufficient study follow-up resulting in data immaturity or simply that data on OS have not been collected. In such instances, determining the impact of health technologies becomes more challenging and may rely on the use of surrogate endpoints to substitute for, and predict, a final patient-relevant clinical outcome.^2^ Potentially relevant surrogate endpoints vary according to tumour type and site, but commonly include progression-free survival (PFS), time to progression (TTP), and response-based outcomes, which may include overall response rate (ORR), different levels of response (e.g. complete response [CR], partial response [PR] or very good partial response [VGPR]) and duration of response (DoR). These surrogate endpoints are often considered attractive as they typically require smaller sample sizes, occur faster and are less expensive to collect in clinical trials compared with final outcomes, thereby reducing costs associated with data collection and expediting the time required for bringing new technologies to market.

It has been recognised in the literature that the reliance on surrogates may lead to invalid conclusions regarding the net health effects of technologies, which in turn have the potential to lead to patient harm.^3^ Much of the published literature around the use of surrogate endpoints has focussed on the development and application of frameworks for their validation.^4,5^ In his seminal paper, Prentice^4^ put forward stringent criteria for the validation of surrogate endpoints in phase 3 trials. In general terms, these criteria require that the surrogate endpoint must be a correlate of the net effect of treatment on the final clinical outcome—in other words, there must be a single pathway from the treatment to the true endpoint, which is mediated exclusively by the surrogate endpoint.^6^ Applied surrogate validation studies commonly adopt a meta-analytic (meta-regression) approach based on multiple studies in order to assess whether the apparent relationship between the surrogate and the final endpoint remains constant in the presence of various sources of heterogeneity, such as differences in patient population, study design and treatments received.^5^

Based on the NIH Biomarkers Definition Working Group’s preferred terms and definitions^7^ and the 2001 *Journal of the American Medical Association* (JAMA) User’s Guide,^8^ Taylor and Elston^9^ proposed a hierarchy of levels of surrogate validation. Level 3 of the hierarchy relates to biological plausibility—this is the weakest form of validation and is typically based on pathophysiological studies and/or an understanding of the disease process. Level 2 requires the presence of a consistent association between the surrogate outcome and the final endpoint; this may be assessed using observational studies or arm-based analyses of trials, which have measured both the surrogate and the final outcome. This level of validation requires an assessment of the individual-level (absolute) association between endpoints and is usually undertaken using correlation analysis. Level 1 of the hierarchy represents the strongest level of surrogate validation: in order to achieve this level of validation, the treatment effect on the surrogate must correspond to the treatment effect on the final outcome. Demonstrating this level of validity requires an analysis of correlation in terms of treatment effects between arms based on data from RCTs (trial-level association). Other validation frameworks have been proposed to assess the strength of association between surrogate and final endpoints. These include the criteria proposed by the German Institute of Quality and Efficiency in Health Care^10^ (IQWiG; based on the treatment effect association only) and the Biomarker-Surrogate Evaluation Schema criteria^11^ (BSES2; based on both absolute and treatment effect associations). These frameworks differ in terms of the types of analyses and the strength of the relationship required to determine the reliability of the surrogate.

This systematic review summarises published meta-regression studies reporting correlation and regression analyses for the strength of the association between response-based outcomes and PFS, TTP or OS in (primarily) advanced or metastatic cancer, across any tumour site, in order to assess whether response-based outcomes may be considered as valid surrogates for PFS, TTP or OS.

## Methods

### Inclusion and exclusion criteria

Inclusion was restricted to articles reporting meta-analyses or meta-regressions across multiple studies and reporting the strength of association between response outcomes (ORR, CR, PR, VGPR or DoR) and either PFS, TTP or OS. The included meta-regressions could themselves include RCTs and/or single-arm studies. However, individual reports analysing single trials or single cohorts were excluded from this review. Included meta-analyses could report absolute associations and/or treatment effect associations. These associations had to be reported as a correlation coefficient (e.g. Pearson’s *r* or Spearman’s *r*_s_) and/or a coefficient of determination (*R*^2^) between relevant outcomes.

Studies of any cancer and any treatment were included. The review focussed mainly on studies of advanced or metastatic cancers (and/or treatment with palliative intent), as these studies were more likely to report PFS and OS. However, studies reporting relevant outcomes were included even where the stage was not specifically restricted to advanced/metastatic disease for all patients or where this was unclear (this applied particularly to haematological cancers). Studies were excluded if they explicitly referred to adjuvant or neo-adjuvant treatment, or treatments that are given with curative intent. Studies were only included if they were written on English or contained sufficient detail in English.

The review protocol is registered on PROSPERO with registration number CRD42019127606.

### Search strategy

Five databases (MEDLINE, EMBASE, Web of Science, the Cochrane Database of Systematic Reviews and CINAHL) were searched from inception to March 2019. Search terms included cancer terms AND response terms AND terms for PFS, TTP and/or OS AND terms for regression, correlation, prediction, association or relationship AND terms for endpoint and/or surrogate. Search results were limited to the English language and to studies undertaken in humans. The MEDLINE search strategy is provided in Supplementary Information 1. In addition, a citation search was undertaken based on two existing meta-reviews of surrogate relationships; this identified studies that have cited any of the 48 articles included in the review by Fischer et al.^12^ and/or any of the 19 articles included in the review by Davis et al.^13^ In addition, relevant existing meta-reviews, including Fischer et al.,^12^ Davis et al.,^13^ Savina et al.,^14^ Haslam et al.^15^ and any reviews identified during searching, were checked for relevant studies.

### Scoring the strength of association: IQWiG and BSES2 scoring

In this review, two sets of published criteria were used to assess the strength of association between surrogate and final endpoints: the IQWiG criteria^10^ and the BSES2 criteria.^11^

The IQWiG criteria^10^ are based on the correlation coefficient (*r*) for the treatment effect association. Where *r* was not reported, it was calculated as the square root of *R*^2^, if available. As the medium score bracket was not clearly defined, slight modifications were made to the IQWiG criteria based on the approach used in the previous review by Savina et al.^14^ (Supplementary Table 1). The IQWiG score was generated based on the magnitude of *r*, irrespective of its sign (i.e. a negative correlation could generate a high score). The IQWiG criteria were scored as follows: high (lower confidence interval of *r* is ≥0.85); medium+ (*r* ≥ 0.85 with no reported confidence interval or *r* ≥ 0.85 with wide confidence intervals [lower limit <0.85]); medium (0.85 > *r* ≥ 0.7 and upper confidence interval of *r* is ≥0.7 and lower confidence interval of *r* is <0.85, or 0.85 > *r* ≥ 0.7 with no reported confidence interval); or low (upper confidence interval of *r* is <0.7 or *r* < 0.7 with no reported confidence interval).

The BSES2 criteria^11^ require *R*^2^ values for both the absolute and treatment effect associations. Where *R*^2^ was not reported, it was calculated as the square of *r*, if available. BSES2 criteria were used as an adaptation from the original BSES criteria, as described in Savina et al.^14^ The original BSES criteria require *R*^2^ for both individual and treatment effect associations and a value for the surrogate threshold effect (STE). Since so few articles report STE, this review used BSES2, which does not require the STE. The BSES2 criteria were scored as follows: excellent (*R*^2^ [treatment effect] ≥0.6 and *R*^2^ [absolute] ≥0.6); good (*R*^2^ [treatment effect] ≥0.4 and *R*^2^ [absolute] ≥0.4); fair (*R*^2^ [treatment effect] ≥0.2 and *R*^2^ [absolute] ≥0.2); poor (*R*^2^ [treatment effect] <0.2 and/or *R*^2^ [absolute] <0.2). Further details on the IQWiG and BSES2 scoring systems are provided in Supplementary Tables 1 and 2.

### Study selection and data extraction

Titles and abstracts of articles retrieved by the search were examined by one reviewer and a subset was checked by a second reviewer early in the process, followed by a discussion to ensure consistency in the selection decisions. Full texts were examined by one reviewer and a subset was checked by a second reviewer, with any discrepancies resolved through discussion.

Data were extracted by one reviewer and all data were checked by a second reviewer. Data were extracted relating to study design, participant characteristics, surrogate and final endpoints analysed, methods for correlation and regression, and results including absolute associations, associations between treatment effects, STE and regression equations.

### Data synthesis

Data were presented in a narrative synthesis. Plots were constructed to illustrate the reported associations within each study. Some of the included meta-regression studies reported multiple subgroup analyses with differing results. Therefore, each horizontal row in the plots illustrates the range of reported associations across all subgroup analyses within a single meta-regression study. Where an included meta-regression study reported on more than one cancer type, these are shown on separate rows on the plots.

For associations between absolute values of endpoints, the plots show the range of correlation coefficients per study, across all subgroup analyses. All types of correlation coefficient were included, for example, Pearson’s *r* and Spearman’s *r*_s_. If no correlation coefficient was reported, then Pearson’s *r* was calculated as the square root of *R*^2^, if available.

For associations between treatment effects, the plots show the range of regression coefficients of determination (*R*^2^) per study, across all subgroup analyses. The plots include both adjusted and unadjusted *R*^2^ values, as well as values from weighted and unweighted regressions. For studies in which *R*^2^ was not reported, this was calculated as the square of the Pearson’s *r* correlation coefficient, if available. *R*^2^ was not calculated from other correlation coefficients such as Spearman, or where the method of correlation was unclear.

### Quality assessment

Included meta-regression studies were assessed for methodological quality based on key criteria from the AMSTAR-2^16^ and ReSEEM^17^ checklists most relevant to our review.

## Results

### Number of included meta-regression studies

**Fig. 1:**
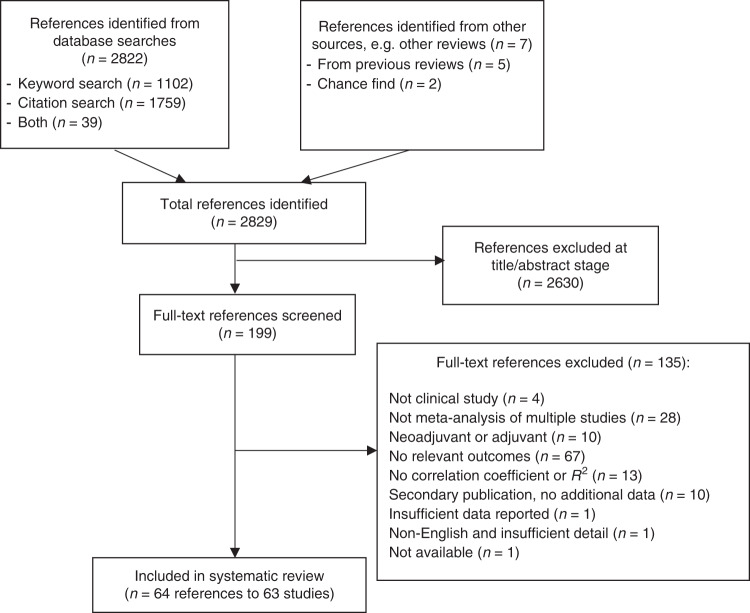
PRISMA flow diagram for study inclusion. Illustrates the number of references retrieved from the literature searches and included/excluded at each stage of screening.

The literature search generated 2829 citations (Fig. 1), of which 2630 were excluded during the review of titles and abstracts and a further 135 excluded during the review of full texts. In total, 63 studies (within 64 references) were included in the review.^18–81^

### Characteristics of included meta-regression studies

Summaries of study characteristics and reported data types are provided in Supplementary Tables 3 and 4, respectively, while full details of study characteristics for each of the 63 included studies are provided in Supplementary Table 5.

The most commonly reported surrogate relationships were ORR to OS (57 studies), ORR to PFS (22 studies), CR to OS (8 studies) and CR to PFS (7 studies). Other response outcomes (DoR, PR and VGPR/CR) were only reported in one to two studies each. Twenty different cancer types were analysed, the most common being NSCLC (16 studies), CRC (10 studies), various solid tumours (8 studies) and breast cancer (5 studies). Disease stage was advanced/metastatic in 43 studies and unclear in 9 studies, while the remainder (11 studies) gave other descriptions mostly indicating advanced, extensive or recurrent disease. Treatment was first line in 23 studies, later lines or combinations of lines in 32 studies and not reported in 8 studies. Treatment type was chemotherapy in 21 studies, immune checkpoint inhibitors in 9 studies, targeted therapy in 8 studies and various other treatment combinations in the remainder.

The various meta-regressions included between 4 and 191 primary studies and between 407 and 44,125 patients each. The majority of meta-regressions (*N* = 44) included only RCTs, while 17 included both RCTs and single-arm studies and 2 included only single-arm studies. Most meta-regressions (*N* = 58) analysed aggregate data (e.g. medians or other summary measures per study arm), while 5 analysed individual patient data (IPD). Across all meta-regressions, 32 reported absolute (individual-level) associations, 38 reported treatment effect (trial-level) associations and only 4 reported the STE.

### Methodological quality of included meta-regression studies

Methodological quality of the included studies is shown in Supplementary Table 6. All studies had clear inclusion criteria; 65% reported a comprehensive literature search; and 98% reported a correlation coefficient or *R*^2^ value (the one study not reporting these was included as it reported a regression slope). However, only 27% reported duplicate study selection; 48% reported duplicate data extraction or checking; and 13% reported a risk of bias assessment of included studies. In addition, only 37% explored heterogeneity through subgroup analyses, and only 40% reported confidence intervals around the correlation coefficient or *R*^2^.

### Results of included studies

**Fig. 2:**
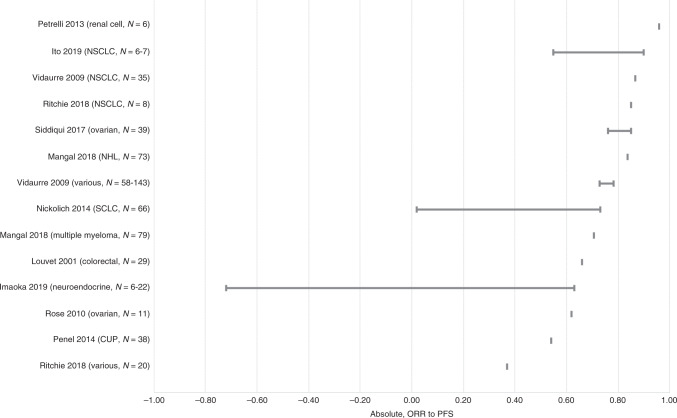
Correlation (*r* or *r*_s_) between absolute (individual-level) values of ORR and PFS. For each study, the plot illustrates the range of correlation coefficients across all subgroup analyses. *N* represents the number of studies included in each meta-regression. CUP cancer of unknown primary, NHL non-Hodgkin’s lymphoma, NSCLC non-small cell lung cancer, ORR overall response rate, PFS progression-free survival, SCLC small cell lung cancer.

**Fig. 3:**
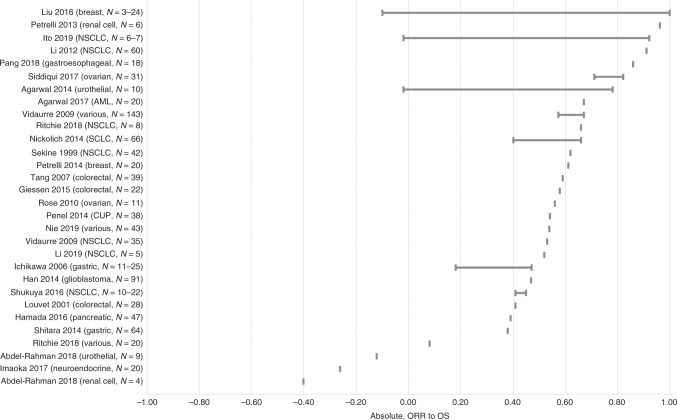
Correlation (*r* or *r*_s_) between absolute (individual-level) values of ORR and OS. For each study, the plot illustrates the range of correlation coefficients across all subgroup analyses. *N* represents the number of studies included in each meta-regression. AML, acute myeloid leukaemia, CUP cancer of unknown primary, NSCLC non-small cell lung cancer, ORR overall response rate, OS overall survival, SCLC small cell lung cancer.

**Fig. 4:**
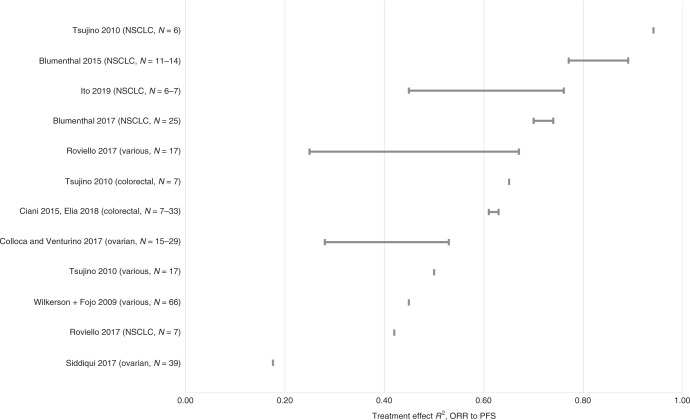
Regression *R*^2^ between treatment effects (trial-level) for ORR and PFS. For each study, the plot illustrates the range of correlation coefficients across all subgroup analyses. *N* represents the number of studies included in each meta-regression. NSCLC non-small cell lung cancer, ORR overall response rate, PFS progression-free survival.

**Fig. 5:**
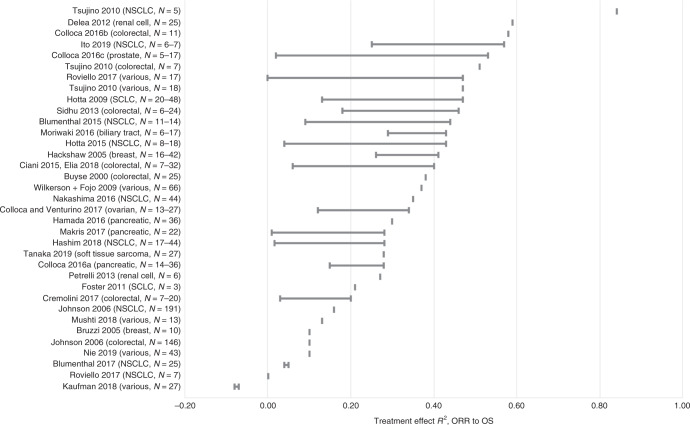
Regression *R*^2^ between treatment effects (trial-level) for ORR and OS. For each study, the plot illustrates the range of correlation coefficients across all subgroup analyses. *N* represents the number of studies included in each meta-regression. NSCLC non-small cell lung cancer, ORR overall response rate, OS overall survival, SCLC small cell lung cancer.

The reported associations between surrogate and final endpoints are summarised in Table 1 and illustrated in Figs. 2–5. Full results for each included meta-regression study are provided in Supplementary Table 7 (for absolute associations) and Supplementary Table 8 (for treatment effect associations).

**Table 1:** Summary of absolute correlation coefficients and treatment effect *R*^2^ values.

Surrogate relationship	Range of absolute (individual-level) correlations			Range of treatment effect (trial-level) *R*^2^ values		
	*N* studies	Cancer types and refs.	Range of *r* or *r*_s_ across studies and subgroup analyses	*N* studies	Cancer types and refs.	Range of *R*^2^ across studies and subgroup analyses
ORR to PFS	12	NSCLC,^45,65,78^ ovarian,^66,72^ RCC,^63^ NHL,^54^ SCLC,^59^ MM,^55^ CRC,^52^ CUP,^62^ NET,^44^ various^65,78^	−0.72 to 0.96	9	NSCLC,^21,22,45,67,77^ ovarian,^27,72^ various,^67,77,79^ CRC^26,77^	0.18 to 0.94
ORR to TTP	1	Gastric^42^	0.41 to 0.56	0		–
ORR to OS	27	NSCLC,^45,49,50,65,68,71,78^ CRC,^35,52,75^ ovarian,^66,72^ breast,^51,64^ gastric,^42,70^ various,^60,65,78^ pancreatic,^37^ RCC,^18,63^ gastroesophageal,^61^ urothelial,^18,19^ AML,^20^ SCLC,^59^ glioblastoma,^38^ CUP,^62^ NET^43^	−0.40 to 1.00	31	NSCLC,^21,22,39,40,45,46,58,67,77^ CRC,^25,26,29,31,46,73,77^ various,^47,57,60,67,77,79^ pancreatic,^28,37,53^ SCLC,^34,41^ RCC,^32,63^ breast,^23,36^ ovarian,^27^ prostate,^30^ BTC,^56^ STC^74^	−0.08 to 0.84
CR to PFS	2	SCLC,^59^ NHL^81^	0.22 to 0.83	1	NHL^69^	0.45 to 0.93
CR to OS	3	NSCLC,^49^ SCLC,^59^ gastroesophageal^61^	−0.04 to 0.62	2	Breast,^36^ SCLC^34^	0.05 to 0.48
PR to PFS	1	SCLC^59^	0.35 to 0.70	0		–
PR to OS	1	SCLC^59^	0.29 to 0.66	0		–
VGPR/CR to PFS	0		–^a^	0		–
DoR to PFS	0		–	0		–
DoR to OS	0		–	0		–^b^

Notes: Further detail on all studies and outcomes is shown in Supplementary Appendixes 5 and 6.

*AML* acute myeloid leukaemia, *BTC* biliary tract cancer, *CR* complete response, *CRC* colorectal cancer, *CUP* cancer of unknown primary, *DoR* duration of response, *MM* multiple myeloma, *NET* neuroendocrine tumour, *NHL* non-Hodgkin’s lymphoma, *NSCLC* non-small cell lung cancer, *ORR* overall response rate, *OS* overall survival, *PFS* progression-free survival, *PR* partial response, *RCC* renal cell carcinoma, *SCLC* small cell lung cancer, *STC* soft tissue sarcoma, *TTP* time to progression, *VGPR* very good partial response.

^a^One study of MM reported the association between VGPR/CR and PFS as adjusted *R*^2^ = 0.64, but this could not be converted to *r* because it was adjusted.^55^

^b^Two studies in CRC^29^ and pancreatic cancer^28^ reported Spearman’s correlation coefficients between DoR and OS ranging from 0.40 to 0.76, but these could not be converted to *R*^2^ as no Pearson’s correlation coefficients were reported.

### Absolute (individual-level) correlation and regression

The range of absolute (individual-level) correlation coefficients is summarised in Table 1 and illustrated in Fig. 2 (for the association between ORR and PFS) and Fig. 3 (for the association between ORR and OS). Some of the included meta-regression studies reported multiple subgroup analyses with differing results. Therefore, each horizontal row in the plots illustrates the range of correlation coefficients across all subgroup analyses within a single meta-regression study. Where an included meta-regression reported on more than one cancer type, these are shown on separate rows on the plots. It is worth noting that the included meta-regression studies differed in terms of various factors, such as the number of included primary studies (shown as *N* on the plots), treatment type, line of treatment and precise clinical population (full details in Supplementary Table 7).

#### ORR and PFS (or TTP)

The reported correlation coefficients (Pearson’s *r* or Spearman’s *r*_s_) between absolute ORR and PFS ranged from −0.72 to 0.96, based on multiple analyses within 12 studies across 10 cancer types^44,45,52,54,55,59,62,63,65,66,72,78^ (Fig. 2 and Table 1). Across those studies that report only a single analysis, the correlation coefficient was generally >0.60; however, some estimates were lower. Confidence intervals around the correlation coefficients were rarely reported. Few separate meta-regressions reported on the same tumour site, hence it is difficult to assess whether ORR may be a more reliable surrogate in certain cancer types than others. One study reported on ORR and TTP (gastric cancer; correlation *r*_s_ = 0.41–0.56 across subgroup analyses, not shown on the plot).^42^

#### ORR and OS

The reported correlation coefficients between absolute ORR and OS ranged from −0.40 to 1.00, based on 27 studies across 15 cancer types^18–20,35,37,38,42,43,45,49–52,59–66,68,70–72,75,78^ (Fig. 3 and Table 1). Confidence intervals around the correlation coefficients, where reported, were generally fairly wide. The majority of correlation coefficients were >0.40; however, several estimates were lower. Neither the correlation coefficients reported from multiple analyses within the same study, nor those reported across separate studies, suggested a clear pattern by cancer type.

#### CR and PFS or OS

The correlation coefficients between absolute CR and PFS in two studies of small cell lung cancer (SCLC)^59^ and non-Hodgkin’s lymphoma (NHL)^81^ ranged from 0.22 to 0.83, while the correlation coefficients between absolute CR and OS ranged from −0.04 to 0.62, based on three studies of NSCLC,^49^ SCLC^59^ and gastroesophageal cancer^61^ (Table 1).

#### PR and PFS or OS

The correlation coefficient between absolute PR and PFS ranged from 0.35 to 0.70 across subgroup analyses within one study of SCLC,^59^ while the highest correlation coefficient between absolute PR and OS ranged from 0.29 to 0.66 in the same study^59^ (Table 1).

#### DoR and PFS or OS

No studies reported on the absolute association between DoR and PFS or OS.

### Treatment effect (trial-level) correlation and regression

The range of treatment effect (trial-level) *R*^2^ values is summarised in Table 1 and illustrated in Fig. 4 (for the association between ORR and PFS) and Fig. 5 (for the association between ORR and OS). Each horizontal row in the plots illustrates the range of *R*^2^ values across all subgroup analyses within a single meta-regression study. Where an included meta-regression reported on more than one cancer type, these are shown separately on the plots. It is worth noting that the meta-regressions differed in terms of the number of included primary studies (shown as *N* on the plots), treatment type, line of treatment and precise clinical population (full details in Supplementary Table 8).

#### ORR and PFS

The regression *R*^2^ values for the treatment effect association between ORR and PFS ranged from 0.18 to 0.94, based on nine studies across five cancer types: NSCLC,^21,22,45,67,77^ ovarian cancer,^27,72^ colorectal cancer^26,77^ and various solid tumours^67,77,79^ (Fig. 4 and Table 1). The majority of *R*^2^ values were above 0.40. The *R*^2^ values reported from multiple analyses within the same study, and those reported across separate studies, did not suggest a clear pattern by cancer type. Confidence intervals around the *R*^2^ values, where reported, were generally fairly wide.

#### ORR and OS

The regression *R*^2^ values for the treatment effect association between ORR and OS ranged from −0.08 to 0.84, based on 31 studies across 11 cancer types^21–23,25–32,34,36,37,39–41,45–47,53,56–58,60,63,67,73,74,77,79^ (Fig. 5 and Table 1). With the exception of one analysis,^77^ all *R*^2^ values were below 0.60. The *R*^2^ values reported from multiple analyses within the same study, and those reported across separate studies, did not suggest a clear pattern by cancer type. Confidence intervals around the *R*^2^ values, where reported, were generally wide.

#### CR and PFS or OS

The regression *R*^2^ for the treatment effect association between CR and PFS ranged from 0.45 to 0.93 across subgroup analyses within one study of NHL,^69^ while the regression *R*^2^ for the treatment effect association between CR and OS within two studies of breast cancer^36^ and SCLC^34^ ranged from 0.05 to 0.48 (Table 1).

#### PR and PFS or OS

No studies reported the treatment effect association between PR and PFS or OS.

#### DoR and PFS or OS

No studies reported *R*^2^ between DoR and OS or PFS. Two studies in colorectal cancer^29^ and pancreatic cancer^28^ reported Spearman’s correlation coefficients between DoR and OS ranging from 0.40 to 0.76 (Table 1).

### Influence of clinical and study factors on association

The impact of the following patient and study factors on the association between ORR and OS was explored: treatment line; treatment type; response criteria; adjustment of OS for crossover and post-progression treatments; and aggregate versus IPD data (Supplementary Table 9). No clear effect on the association between ORR and OS was identified for any individual factor. However, this analysis was limited by the small number of publications assessing each factor within each cancer, and the wide ranges of associations observed for each.

Five of the 63 included meta-analyses analysed IPD rather than aggregate data; two in breast cancer^23,24^), one in colorectal cancer^25^, one in NHL^69^ and one in ovarian cancer^66^. The associations reported in these studies were not noticeably different to those in other studies (see Figs. 2–5).

### Regression equations

Regression equations were reported in 14 studies for the relationship between ORR and OS; of these, four reported absolute associations^42,52,72,76^ and ten reported treatment effect associations.^31–33,36,41,46,56,58,67,77^ Regression equations were also reported in eight studies for the relationship between ORR and PFS; of these, four reported absolute associations^52,54,72,76^ and four reported treatment effect associations.^24,33,67,77^ These analyses spanned 10 cancer types. Full details are provided in Supplementary Tables 10 and 11. There was substantial variation in the effect measures used for both the surrogate and final outcomes; for example, difference in medians, hazard ratio (HR), odds ratio (OR), log-transformed or not. None of the included studies attempted to externally validate their regression equations for the relationship between response and other outcomes.

### Surrogate threshold effect

The STE—the smallest treatment effect on the surrogate that predicts a non-zero treatment effect on the true endpoint^82^—was reported in only four studies (Supplementary Table 12).^26,39,69,77^ For the relationship between ORR and PFS, one study^77^ in various solid tumours reported that a difference in ORR of 15% would be required to predict a non-zero treatment effect on the HR for PFS. For the relationship between ORR and OS, two studies in various solid tumours^77^ and NSCLC^39^ reported that a difference in ORR of 21% and 55%, respectively, would be required to predict a non-zero treatment effect on the HR for OS, while one study^39^ also reported that a difference in ORR of 41% would be required to predict a non-zero treatment effect on the difference in median OS. A further study in colorectal cancer^26^ reported that an OR for ORR of 0.28 would be required to predict a non-zero treatment effect on the OR for OS. Finally, for the relationship between CR and PFS, one study in NHL^69^ reported that an OR for CR (at 30 months) of 1.56 would be required to predict a non-zero treatment effect on the HR for PFS.

### IQWiG and BSES2 scores for the strength of association

IQWiG and BSES2 scores for the strength of association between surrogate and final endpoints were calculated for all reported subgroup analyses with sufficient data; therefore, meta-regression studies that reported more subgroups are more strongly represented in this analysis. These data are presented graphically in Supplementary Figs. 1 and 2.

In terms of IQWiG scores, of 202 analyses (across 63 studies), 0 (0%) scored high, 15 (7%) scored medium+, 26 (13%) scored medium, 76 (38%) scored low and 85 (42%) were not evaluable. In terms of BSES2 scores, of 202 analyses (across 63 studies), 0 (0%) scored excellent, 3 (1%) scored good, 3 (1%) scored fair, 7 (3%) scored poor and 189 (94%) were not evaluable.

## Discussion

This systematic review summarises published meta-regression studies reporting correlation and regression analyses for the strength of the association between response outcomes and PFS, TTP or OS across different types of cancer. In total, the review included 63 studies across 20 cancer types. The most commonly analysed relationships were between ORR and either PFS or OS.

For the association between ORR and PFS, the majority of reported correlation coefficients between absolute values were >0.60 (range −0.72 to 0.96). For association between treatment effects on ORR and PFS, the majority of regression *R*^2^ values were >0.40 (range 0.18–0.94). The association between ORR and OS appeared weaker than that between ORR and PFS; while the majority of reported correlation coefficients between absolute values were >0.40, several estimates were lower (range −0.40 to 1.00). For association between treatment effects on ORR and OS, all regression *R*^2^ values except one were below 0.60 (range −0.08 to 0.84).

There was no clear pattern by cancer type for either the absolute or treatment effect associations, based on both multiple analyses within the same study and results across separate studies. Confidence intervals around the reported correlation coefficients and R^2^ values were generally wide and often not reported.

Strength of association across all subgroup analyses within all included meta-regression studies was assessed using the IQWiG and BSES2 scoring systems. The majority of analyses were not evaluable due to the lack of required data. Of those analyses that could be scored, scores were relatively low, suggesting that response-based endpoints may be poor surrogates for OS.

Previous systematic reviews of surrogate endpoints in advanced cancer have been published. Savina et al.^14^ and Haslam et al.^15^ have reported systematic reviews of meta-analyses assessing any endpoint as a surrogate for OS. Both these reviews also assessed the strength of association using surrogate validation frameworks; both studies used adaptations of the IQwiG framework, and Savina et al.^14^ also used the BSES2 framework. These previous reviews generally focussed on the main analyses presented within individual meta-analyses (usually that with the largest number of patients). Similar to our review, these previous reviews suggested that response-based outcomes are likely to be poor surrogates for OS. Our systematic review focusses exclusively on response-based surrogates; it includes a comprehensive search to identify relevant studies, considers PFS as a potential final endpoint as well as OS, is more up to date, includes a greater number of studies and reports results for the full breadth of analyses reported in the included meta-regression studies compared with these previous reviews. This provides a more complete picture of the extent of heterogeneity in reported relationships across the full range of meta-analyses across each cancer area. This additional breadth provides a better basis to inform judgements about the validity of response-based endpoints as a surrogate for PFS or OS.

The review is subject to a number of limitations. The reported data were highly heterogeneous in terms of effect measure and method of analysis. Therefore, some simplifying assumptions had to be made to allow the data to be summarised. For example, correlation coefficients were summarised regardless of method (Pearson’s, Spearman’s or other); *R*^2^ values were summarised irrespective of whether or not the regression was weighted and whether or not the *R*^2^ was adjusted; and for treatment effect associations, *R*^2^ values were summarised regardless of the effect measure (e.g. HR, OR, difference in medians). In addition, only five studies used IPD rather than aggregate data in their analysis; this is a limitation of the analyses conducted in the majority of meta-reviews. A recent review by Xie et al.^17^ highlighted wide variability in reporting standards across surrogate evaluation meta-regression studies; future analyses should attempt to adhere to current best practice, for example, the reporting of surrogate endpoint evaluation using meta-analyses (ReSEEM) guidelines in order to improve the quality of these analyses.^17^

It should further be noted that while meta-regression has been widely used for the purpose of evaluating the validity of surrogate endpoints in oncology, this method has been criticised as it ignores uncertainty around the treatment effect on the surrogate outcome (which is treated as a fixed covariate in the analysis). Newer methods, such as the bivariate random effects meta-analysis (BRMA) model reported by Bujkiewicz et al.,^83^ provides an approach for both the validation and prediction of surrogate endpoints within a Bayesian framework. This approach allows for borrowing of information across studies and fully accounts for all uncertainty surrounding the surrogate relationship. In spite of the generally poor association between response-based outcomes and final outcomes, there may still be instances in which generating predictions on the basis of response is necessary; for example, within health economic models, or more broadly, for decision-making within health technology assessment. In instances where the surrogate association is weak, this uncertainty would manifest as a wider prediction interval. If such predictions are necessary, it is therefore important that all uncertainty is reflected in the model. Future surrogate evaluation studies should consider the use of the BRMA model, rather than conventional meta-regression, as a means of fully reflecting this uncertainty.

## Conclusions

This systematic review suggests that response-based endpoints such as ORR and CR may not be reliable surrogates for PFS or OS in cancer treatment. Strength of association varied widely between and within studies, with no clear pattern by cancer type. The strength of association between ORR and OS appeared weaker and more variable than that between ORR and PFS, both for associations between absolute endpoints and associations between treatment effects. While there may still be value in using response outcomes as a means of predicting final outcomes such as OS, it is important that those predictions are made on the basis of models which fully reflect the uncertainty around the treatment effect on the surrogate outcome.

## Supplementary information

Supplementary Information

## Data Availability

All data are provided in the tables, figures and supplementary information.
