# Supplementary material for: A systematic review of meta-analyses assessing the validity of tumour response endpoints as surrogates for progression-free or overall survival in cancer
Source: Br J Cancer. 2020 Sep 11;123(11):1686–96. doi: 10.1038/s41416-020-01050-w (PMC7687906; doi:10.1038/s41416-020-01050-w)

**A systematic review of meta-analyses assessing the validity of tumour response endpoints as surrogates for progression-free or overall survival in cancer**

Katy Cooper, Paul Tappenden, Anna Cantrell**,** Kate Ennis

| **Supplementary Information** | **Page** |
| --- | --- |
| Supplementary Information 1: MEDLINE search strategy | 2 |
| Supplementary Table 1: IQWiG scoring criteria | 3 |
| Supplementary Table 2: BSES2 scoring criteria | 3 |
| Supplementary Table 3: Summary of study characteristics | 4 |
| Supplementary Table 4: Summary of reported data types | 4 |
| Supplementary Table 5: Study characteristics by study | 5 |
| Supplementary Table 6: Quality assessment of included meta-reviews | 11 |
| Supplementary Table 7: Absolute correlation and regression results per study | 14 |
| Supplementary Table 8: Treatment effect correlation and regression results per study | 22 |
| Supplementary Table 9: Influence of clinical and study factors on association between ORR and OS | 36 |
| Supplementary Table 10: Regression equations for absolute (individual-level) associations | 40 |
| Supplementary Table 11: Regression equations for treatment effect (trial-level) associations | 41 |
| Supplementary Table 12: Surrogate threshold effect (STE) data reported per study | 43 |
| Supplementary Figure 1: IQWiG scores for strength of association across all 202 analyses (within 63 included studies) | 44 |
| Supplementary Figure 2: BSES2 scores for strength of association across all 202 analyses (within 63 included studies) | 45 |

Supplementary Information 1: MEDLINE search strategy

Search Strategy (March 2019):

1 *Neoplasms/

2 (cancer$ or neoplasm$ or tumour$ or tumour$ or malignan$ or oncology or lymphoma$ or sarcoma$ or melanoma$ or myeloma$ or carcinoma$).tw.

3 1 or 2

4 tumour response$.tw.

5 tumour response$.tw.

6 objective response$.tw.

7 ORR.tw.

8 "duration of response$".tw.

9 dor.tw.

10 response rate$.tw.

11 complete response$.tw

12 overall response$.tw

13 4 or 5 or 6 or 7 or 8 or 9 or 10 or 11 or 12

14 3 and 13

15 Regression analysis/

16 regression.tw.

17 relationship.tw.

18 correlation.tw.

19 prediction.tw.

20 association.tw.

21 15 or 16 or 17 or 18 or 19 or 20

22 14 and 21

23 endpoint$.tw.

24 end point$.tw.

25 (surrogate or surrogacy).tw.

26 23 or 24 or 25

27 22 and 26

28 progression-free survival/

29 "progression free survival".tw.

30 "overall survival".tw.

31 (pfs or os).tw.

32 "time to progression".tw.

33 ttp.tw.

34 28 or 29 or 30 or 31 or 32 or 33

35 27 and 34

36 limit 35 to (english language and humans)

Supplementary Table 1: IQWiG scoring criteria

| **IQWiG Score** | **Criteria (based on r for treatment-effect association)*** |
| --- | --- |
| High | Lower confidence interval of r is ≥ 0.85 |
| Medium+ | r ≥ 0.85 with no reported confidence interval **or** r ≥ 0.85 with wide confidence intervals (lower limit <0.85) |
| Medium | 0.85 > r ≥ 0.7 and upper confidence interval of r is ≥ 0.7 and lower confidence interval of r is < 0.85, **or** 0.85 > r ≥ 0.7 with no reported confidence interval |
| Low | Upper confidence interval of r is < 0.7 **or** r < 0.7 with no reported confidence interval |
| Notes:  Based on the scoring criteria reported by IQWiG (2011).^10^  *r is defined as any correlation parameter for the treatment-effect association, e.g. Pearson, Spearman, Kendall's Tau. Where no correlation parameter was reported, if a univariate regression was performed and an R^2^ value attained, then r (Pearson correlation coefficient) was calculated as the square-root of R^2^. The reported r could be for any treatment effect estimate (hazard ratio, difference in medians, etc.); where more than one was reported, relative estimates (e.g. hazard ratio, odds ratio) were used in preference to difference in medians. The Medium+ category was based on the approach used in Savina *et al*.^14^ | |

Supplementary Table 2: BSES2 scoring criteria

| **BSES2 score** | **Criteria (based on R^2^ for both treatment effect and individual-level associations)*** |
| --- | --- |
| Excellent | R^2^ (treatment effect) ≥ 0.6 and R^2^ (absolute) ≥ 0.6 |
| Good | R^2^ (treatment effect) ≥ 0.4 and R^2^ (absolute) ≥ 0.4 |
| Fair | R^2^ (treatment effect) ≥ 0.2 and R^2^ (absolute) ≥ 0.2 |
| Poor | R^2^ (treatment effect) < 0.2 and/or R^2^ (absolute) < 0.2 |
| Notes:  Based on the scoring criteria reported by Lassere et al. (2012).^11^  *R^2^ is the coefficient of determination for a regression analysis. Where R^2^ was not reported, it was calculated as the square of the Pearson correlation coefficient (r), if available. The reported R^2^ could be for any treatment effect estimate (hazard ratio, difference in medians, etc.); where more than one was reported, relative estimates (e.g. hazard ratio, odds ratio) were used in preference to difference in medians. | |

Supplementary Table 3: Summary of study characteristics

| **Surrogate relationship** | **N** | **Cancer type** | **N** | **Disease stage** | **N** | **Line of treatment** | **N** | **Treatment type** | **N** |  |
| --- | --- | --- | --- | --- | --- | --- | --- | --- | --- | --- |
| ORR to OS  ORR to PFS  CR to OS  CR to PFS  DoR to OS  ORR to TTP  PR to PFS  PR to OS  VGPR/CR to PFS  DoR to PFS | 57  22  8  7  2  1  1  1  1  1 | Lung (NSCLC)  Colorectal  Various solid  Breast  NHL  Lung (SCLC)  Ovarian  Pancreatic  Renal cell  Gastric  Neuroendocrine  Soft tissue sarcoma  Urothelial  AML  Biliary tract  Gastroesophageal  Glioblastoma  Multiple myeloma  Prostate  Unknown primary | 16  10  8  5  4  3  3  3  3  2  2  2  2  1  1  1  1  1  1  1 | Advanced/metastatic  Unclear  Advanced, locally advanced, unresectable or metastatic  Extensive disease  Limited or extensive disease  Advanced or recurrent  Advanced, locally advanced or recurrent  Relapsed / refractory  Most stage III/IV  Recurrent / platinum-resistant  Various | 43  9  2  2  1  1  1  1  1  1  1 | 1st  All / various  NR  1st + 2nd  2nd  2nd + subsequent  2nd + 3rd | 23  18  8  5  4  3  2 | Chemo  Immune checkpoint inhibitors  Targeted  Various  Systemic  Chemo or targeted  Chemo, immune or targeted  NR  Chemo + targeted  Chemo or immune  Chemo, hormonal + targeted  Chemo or biologic  Cytokine or targeted  Gemcitabine + chemo or targeted  Bevacizumab + chemo | 21  9  8  7  5  3  2  1  1  1  1  1  1  1  1 |  |
| Note: Ns may sum to more than total number of studies (N=63) as some studies reported more than one surrogate relationship or cancer type.  AML, acute myeloid leukaemia; chemo, chemotherapy; CR, complete response; DoR, duration of response; immune, immunotherapy; NR, not reported; NSCLC, non-small cell lung cancer; ORR, overall response rate; OS, overall survival; PFS, progression-free survival; PR, partial response; SCLC, small cell lung cancer; TTP, time to progression; VGPR, very good partial response. | | | | | | | | | | |

Supplementary Table 4: Summary of reported data types

| **N primary studies per meta-regression (range)** | **N patients per meta-regression (range)** | **Included study types per meta-regression** | **Data types** | **Absolute association reported?** | **Treatment effect association reported?** | **STE reported?** |
| --- | --- | --- | --- | --- | --- | --- |
| 4 to 191 | 407 to 44,125 | RCT only (N=44)  RCT+SA (N=17)  SA only (N=2) | AD (N=58)  IPD (N=5) | N=32 | N=38 | N=4 |
| AD, aggregate data; IPD, individual patient data; RCT, randomised controlled trials; SA, single-arm studies; STE, surrogate threshold effect. | | | | | | |

Supplementary Table 5: Study characteristics by study

| **Reference** | **Cancer** | **Surrogate outcome** | **Final outcome** | **Stage** | **Line** | **Treatment** | **N studies** | **N patients** | **Study types** | **Publication/ search years** | **Data type** | **Response criteria** | **Absolute association** | **Treatment effect association** | **STE reported** |
| --- | --- | --- | --- | --- | --- | --- | --- | --- | --- | --- | --- | --- | --- | --- | --- |
| Agarwal 2017^20^ | Acute myeloid leukemia | ORR  CR | OS | Various | 1st | Systemic | 20^†^ | NR | RCT + SA | 2004-2016 | AD | NR | Y |  |  |
| Moriwaki 2016^56^ | Biliary tract | ORR | OS | Advanced | 1st | Chemo | 17^†^ | 2040 | RCT | Up to 2015 | AD | NR |  | Y |  |
| Bruzzi 2005^23^ | Breast | ORR | OS | Metastatic | All | Chemo | 10 | 2126 | RCT | 1991-2001 | IPD | WHO (8), ECOG (1), NR (1) |  | Y |  |
| Burzykowski 2008^24^ | Breast | ORR | PFS  OS | Metastatic | 1st | Chemo | 11 | 3953 | RCT | 1999-2008 | IPD | WHO |  | Y |  |
| Hackshaw 2005^36^ | Breast | ORR  CR | OS | Metastatic | 1st | Chemo | 42* | 9163 | RCT | 1966-2005 | AD | NR |  | Y |  |
| Liu 2016^51^ | Breast | ORR* | OS | Metastatic | 2nd + 3rd | Chemo | 24 | 8617 | RCT | 1999 to 2014 | AD | NR | Y |  |  |
| Petrelli 2014^64^ | Breast | ORR | OS | Metastatic or advanced | 1st | Targeted + chemo | 20^†^ | 10138^†^ | RCT | 2000 to 2012 | AD | NR | Y |  |  |
| Buyse 2000^25^ | Colorectal | ORR | OS | Advanced | 1st | Chemo | 25 | 3791 | RCT | Collected 1990-1996 | IPD | WHO |  | Y |  |
| Ciani 2015^26^ Elia 2018^33^ | Colorectal | ORR | PFS  OS | Advanced or metastatic | All | Systemic | 33 | NR | RCT | 2003-2013 | AD | RECIST or WHO |  | Y | Y |
| Colloca 2016b^29^ | Colorectal | ORR  DoR | OS | Metastatic | 1st | Bevacizumab + chemo | 11 | NR | RCT | 2000-2014 | AD | RECIST |  | Y |  |
| Giessen 2015^35^ | Colorectal | ORR | OS | Metastatic | 2nd | Chemo | 22 | 10509 | RCT | 2000-2013 | AD | RECIST (17), WHO (5) | Y |  |  |
| Cremolini 2017^31^ | Colorectal | ORR | OS | Metastatic | 2nd | Targeted | 20* | 7571 | RCT | To 2015 | AD | NR |  | Y |  |
| Johnson 2006^46^ | Colorectal | ORR | OS | Metastatic | 1st | Chemo | 146^†^ | 35337^†^ | RCT | To 2005 | AD | NR (very few RECIST) |  | Y |  |
| Louvet 2001^52^ | Colorectal | ORR | PFS  OS | Metastatic | 1st | Various | 29 | 13498 | RCT | 1990 to 2000 | AD | NR | Y |  |  |
| Sidhu 2013^73^ | Colorectal | ORR | OS | Metastatic | 1st (most) | Chemo +/- targeted | 24^†^ | 20438^†^ | RCT | 2000 to 2011 | AD | NR |  | Y |  |
| Tang 2007^75^ | Colorectal | ORR | OS | Metastatic | 1st | Chemo | 39 | 18668 | RCT | 1990 to 2005 | AD | NR | Y | Y |  |
| Tsujino 2010^77^ | Colorectal | ORR | PFS  OS | Advanced | NR | Targeted | 7 | NR | RCT | Up to 2009 | AD | NR |  | Y |  |
| Ichikawa 2006^42^ | Gastric | ORR | TTP  OS | Advanced | 1st | Chemo | 25 | 4593 | RCT | NR | AD | WHO, SWOG, RECIST, Japan | Y |  |  |
| Shitara 2014^70^ | Gastric | ORR* | OS | Advanced | 2nd + 3rd | Chemo | 64 | 4286 | RCT + SA | 2002 to 2012/2013 | AD | NR | Y |  |  |
| Pang 2018^61^ | Gastroesophageal | ORR*  CR | OS | Advanced | 1st + 2nd | Targeted | 18 | 7892 | RCT | Up to 2018 | AD | RECIST | Y |  |  |
| Han 2014^38^ | Glioblastoma | ORR | OS | Unclear | Various | Various | 91^†^ | 7125† | RCT + SA | 1991-2012 | AD | NR ("standard criteria") | Y |  |  |
| Blumenthal 2017^22^ | Lung (NSCLC) | ORR | PFS  OS | Metastatic | Various | Chemo, immune or targeted | 25 | 20013^†^ | RCT | 2003-2016 | AD | RECIST or WHO |  | Y |  |
| Blumenthal 2015^21^ | Lung (NSCLC) | ORR | PFS  OS | Metastatic | Various | Chemo or targeted | 14 | 12567^†^ | RCT | 2003-2013 | AD | RECIST (11) or WHO (3) |  | Y |  |
| Hashim 2018^39^ | Lung (NSCLC) | ORR | OS | Advanced | 2nd + subsequent | Various | 140 | 41725 | RCT | To 2016 | AD | NR |  | Y | Y |
| Hotta 2015^40^ | Lung (NSCLC) | ORR | OS | Advanced | Various | Targeted | 18 | 7633^†^ | RCT | 2003-2014 | AD | NR |  | Y |  |
| Ito 2019^45^ | Lung (NSCLC) | ORR | PFS  OS | Advanced | Various | Immune checkpoint inhibitors (PD-(L)1) | 7 | 3752^†^ | RCT | NR | AD | NR | Y | Y |  |
| Johnson 2006^46^ | Lung (NSCLC) | ORR | OS | Advanced | 1st | Chemo | 191^†^ | 44125^†^ | RCT | To 2005 | AD | NR (very few RECIST) |  | Y |  |
| Li 2019^49^ | Lung (NSCLC) | ORR*  CR | OS | Advanced | 1st + 2nd | Immune checkpoint inhibitors | 5^†^ | 4803^†^ | RCT | Up to 2018 | AD | RECIST | Y |  |  |
| Li 2012^50^ | Lung (NSCLC) | ORR | OS | Advanced | 1st + 2nd | Targeted | 60 | 9903 | RCT + SA | Up to 2011 | AD | RECIST (52), WHO (10) | Y |  |  |
| Nakashima 2016^58^ | Lung (NSCLC) | ORR | OS | Advanced, locally advanced and recurrent | 1st | Chemo | 44 | 22709 | RCT | 2005 to 2015 | AD | RECIST |  | Y |  |
| Ritchie 2018^65^ | Lung (NSCLC) | ORR* | PFS  OS | Advanced | All | Immune checkpoint inhibitors (PD-(L)1 or CTLA4) | 8 | NR | RCT | 2000 to 2017 | AD | NR | Y | Y |  |
| Roviello 2017^67^ | Lung (NSCLC) | ORR | PFS  OS | Unclear | Various | Immune checkpoint inhibitors | 7* | 3369* | RCT | Up to 2017 | AD | RECIST or mWHO |  | Y |  |
| Sekine 1999^68^ | Lung (NSCLC) | ORR | OS | Unclear | Various | Chemo | 42 | 1935 | SA +1 RCT | 1988-1997 | AD | WHO | Y |  |  |
| Shukuya 2016^71^ | Lung (NSCLC) | ORR | OS | Advanced | All | a) Immune checkpoint inhibitors (PD-(L)1)  b) Chemo (docetaxel) | a) 10^†^  b) 22^†^ | NR | RCT + SA | 2012 to 2016 | AD | RECIST (most) | Y |  |  |
| Tsujino 2010^77^ | Lung (NSCLC) | ORR | PFS  OS | Advanced | NR | Targeted | 6 | NR | RCT | Up to 2009 | AD | NR |  | Y |  |
| Tsujino 2009^76^ | Lung (NSCLC) | ORR | PFS  OS | Advanced | NR | Targeted | 28 | 6171 | RCT + SA | To 2007 | AD | RECIST (21), WHO (9) | Y |  |  |
| Vidaurre 2009^78^ | Lung (NSCLC) | ORR* | PFS  OS | Advanced, locally advanced, unresectable or metastatic | NR | Chemo or targeted | 35 | NR | RCT + SA | 2006 to 2008 | AD | NR | Y |  |  |
| Foster 2011^34^ | Lung (SCLC) | ORR  CR | OS | Extensive-stage | 1st | Chemo | 3 RCTs (32 centres) | 596^†^ | RCT | Trials initiated 1987-1999 | AD | NR (CR=disappearance; PR ≥50% reduction |  | Y |  |
| Hotta 2009^41^ | Lung (SCLC) | ORR | OS | Extensive disease | 1st | Chemo | 48 | 8779 | RCT | 1990-2008 | AD | WHO (23), ECOG (2), RECIST (1), Japan (1), or NR |  | Y |  |
| Nickolich 2014^59^ | Lung (SCLC) | ORR  CR  PR | PFS  OS | Limited or extensive disease | 1st + 2nd + maintenance | Various | 66^†^ | 8471^†^ | RCT + SA | 1983 to 2010 | AD | NR | Y |  |  |
| Mangal 2018^55^ (myeloma) | Multiple myeloma | ORR*  CR  VGPR or CR | PFS | Relapsed / refractory | 2nd + subsequent | Various | 79^†^ | 13322^†^ | RCT + SA | 1999 to 2016 | AD | IMWG | Y |  |  |
| Imaoka 2019^44^ | Neuroendocrine | ORR | PFS | Advanced | Various | Systemic | 22 | 1310 | RCT + SA | 1996-2016 | AD | RECIST (20), WHO (2) | Y |  |  |
| Imaoka 2017^43^ | Neuroendocrine | ORR | OS | Advanced | Various | Systemic | 20 | 2530 | RCT + SA | 1996-2016 | AD | NR | Y |  |  |
| Lee 2011^48^ | NHL (aggressive) | CR | PFS  OS | Unclear | 1st | Chemo | 36^†^ | 16103^†^ | RCT | 1990-2009 | AD | NR |  | Y |  |
| Lee 2011^48^ | NHL (indolent) | CR | PFS  OS | Unclear | 1st | Chemo | 15^†^ | 5128^†^ | RCT | 1990-2009 | AD | NR |  | Y |  |
| Mangal 2018^54^ (NHL) | NHL | ORR*  CR | PFS | Stage III/IV >75% in most cohorts | Various | Various | 73 | 6071 | RCT + SA | 1996 to 2015 | AD | NR | Y |  |  |
| Shi 2017^69^ | NHL (indolent; follicular) | CR 30mo  CR 24mo | PFS | Unclear | 1st | Chemo or immuno (induction or maintenance) | 13 | 3837 | RCT | 1990 to 2011 | IPD | NR (CR= disappearance) |  | Y | Y |
| Zhu 2017^81^ | NHL (indolent; follicular) | CR | PFS | Unclear | NR | Chemo, immune or targeted | 13 | NR | RCT + SA | 1993 to 2013 | AD | NR | Y |  |  |
| Zhu 2017^81^ | NHL (mantle cell) | CR | PFS | Unclear | NR | Chemo, immune or targeted | NR | NR | RCT + SA | 1993 to 2013 | AD | NR | Y |  |  |
| Colloca & Venturino 2017^27^ | Ovarian | ORR  CR | PFS  OS | Advanced | 1st | Chemo | 29 | NR | RCT | 1990-2016 | AD | WHO (24), RECIST (8) |  | Y |  |
| Rose 2010^66^ | Ovarian | ORR* | PFS  OS | Recurrent / platinum-resistant | 2nd | Various | 11 | 407 | SA | 1994 to 2004 | IPD | WHO (10), RECIST (1) | Y |  |  |
| Siddiqui 2017^72^ | Ovarian | ORR* | PFS  OS | Advanced, recurrent | 2nd + subsequent | Chemo | 39^†^ | 9223^†^ | RCT | 2000 to 2015 | AD | NR | Y | Y |  |
| Colloca 2016a^28^ | Pancreatic | ORR  DoR | PFS  OS | Advanced or metastatic | 1st | Gemcitabine + chemo or targeted | 36* | NR | RCT | 1997-2014 | AD | RECIST |  | Y |  |
| Hamada 2016^37^ | Pancreatic | ORR | OS | Advanced | 1st | Chemo | 47 | 15906^†^ | RCT | 1995-2015 | AD | NR | Y | Y |  |
| Makris 2017^53^ | Pancreatic (adenocarcinoma) | ORR | OS | Locally advanced, unresectable or metastatic | 1st | Chemo (gemcitabine) | 22* | 10379* | RCT | 2000 to 2015 | AD | NR (RR=shrinkage or disappearance) |  | Y |  |
| Colloca 2016c^30^ | Prostate | ORR | OS | Metastatic (castration-resistant) | 1st + 2nd | Chemo, hormonal + targeted | 17 | NR | RCT | 1995-2014 | AD | NR (CR=disappearance; PR=≥30% reduction) |  | Y |  |
| Abdel-Rahman 2018^18^ | Renal cell | ORR | OS | Advanced | Various | Immune checkpoint inhibitors (PD-(L)1) | 4 | 1093 | RCT + SA | To 2017 | AD | RECIST | Y |  |  |
| Delea 2012^32^ | Renal cell | ORR | OS | Metastatic | NR | Cytokine or targeted | 25* | 10943^†^ | RCT | 1997-2010 | AD | NR |  | Y |  |
| Petrelli 2013^63^ | Renal cell | ORR | PFS  OS | Metastatic | 1st | Targeted | 6^†^ | 3188^†^ | RCT | Up to 2011 | AD | NR | Y | Y |  |
| Tanaka 2019^74^ | Soft tissue sarcoma | ORR | OS | Advanced | 1st | Chemo | 27^†^ | 6156^†^ | RCT | 1974 to 2017 | AD | NR |  | Y |  |
| Zer 2016^80^ | Soft tissue sarcoma | ORR | OS | Advanced or metastatic | All | Systemic | 52^†^ | 9762^†^ | RCT | 1974 to 2014 | AD | NR |  | Y |  |
| Penel 2014^62^ | Unknown primary | ORR* | PFS  OS | Unclear | NR | NR | 38^†^ | NR | SA | 1997 to 2011 | AD | RECIST or WHO | Y |  |  |
| Abdel-Rahman 2018^18^ | Urothelial | ORR | OS | Advanced | Various | Immune checkpoint inhibitors (PD-(L)1) | 9 | 1699 | RCT + SA | To 2017 | AD | RECIST | Y |  |  |
| Agarwal 2014^19^ | Urothelial | ORR | OS | Advanced (operable or metastatic) | 2nd | Chemo or biologic | 10 | 560 | RCT + SA | NR | AD | RECIST | Y |  |  |
| Kaufman 2018^47^ | Various solid tumours | ORR | OS | Unclear | Various | Immune checkpoint inhibitors +/- chemo | 27^†^ | 10300^†^ | RCT | 2005-2017 | AD | RECIST or mWHO |  | Y |  |
| Mushti 2018^57^ | Various solid tumours | ORR* | OS | Unclear | NR | Immune checkpoint inhibitors (PD-(L)1) | 13 | 6722 | RCT | 2014 to 2016 | AD | RECIST |  | Y |  |
| Nie 2019^60^ | Various solid tumours | ORR* | OS | Advanced or recurrent | Various | Immune checkpoint inhibitors (PD-(L)1) | 43^†^ | 15088^†^ | RCT + SA | Up to 2018 | AD | RECIST | Y | Y |  |
| Ritchie 2018^65^ | Various solid tumours | ORR* | PFS  OS | Advanced | All | Immune checkpoint inhibitors (PD-(L)1 or CTLA4) | 20^†^ | 10828^†^ | RCT | 2000 to 2017 | AD | NR | Y | Y |  |
| Roviello 2017^67^ | Various solid tumours | ORR | PFS  OS | Unclear | Various | Immune checkpoint inhibitors | 17^†^ | 8994^†^ | RCT | Up to 2017 | AD | RECIST or mWHO |  | Y |  |
| Tsujino 2010^77^ | Various solid tumours | ORR | PFS  OS | Advanced | NR | Targeted | 18 | NR | RCT | Up to 2009 | AD | NR |  | Y | Y |
| Vidaurre 2009^78^ | Various | ORR* | PFS  OS | Advanced, locally advanced, unresectable or metastatic | NR | Chemo or targeted | 143^†^ | 6974^†^ | RCT + SA | 2006 to 2008 | AD | NR | Y |  |  |
| Wilkerson+Fojo 2009^79^ | Various solid tumours | ORR | PFS  OS | Metastatic | NR | NR | 66^†^ | NR | RCT | NR | AD | NR |  | Y |  |
| Note: Of the 63 included studies (64 refs), 8 references^18,46,48,65,67,77,78,81^ appear on 2-3 rows as they report on 2-3 different cancer types. *Calculated from reported data. ^†^Unclear for individual subgroups.  AD, aggregate data; chemo, chemotherapy; CR, complete response; DoR, duration of response; ECOG, Eastern Cooperative Oncology Group; IMWG, International Myeloma Working Group (criteria); IPD, individual patient data; mo, months; mWHO, modified World Health Organisation (criteria); NHL, non-Hodgkin’s lymphoma; NR, not reported; NSCLC, non-small cell lung cancer; ORR, overall response rate (ORR=PR+CR); OS, overall survival; PFS, progression-free survival; PR, partial response; RCT, randomised controlled trials; RECIST, Response Evaluation Criteria In Solid Tumours; SA, single-arm studies; SCLC, small cell lung cancer; STE, surrogate threshold effect; TTP, time to progression; VGPR, very good partial response; WHO, World Health Organisation (criteria). | | | | | | | | | | | | | | | |

Supplementary Table 6: Quality assessment of included meta-reviews

| **Reference** | **Cancer** | **Inclusion criteria clear & relevant (population, outcomes, study type)** | **Literature search comprehensive (at least 2 databases or other sources AND keywords provided)** | **Duplicate study selection (all or a sample)** | **Duplicate data extraction or data checking** | **Risk of bias assessment reported** | **Analysis methods appropriate: correlation coefficient (r or rs) and/or coefficient of determination (R2)** | **Heterogeneity explored through subgroup analyses** | **Uncertainty assessed (reports 95% confidence intervals for r, rs or R2)** |  |
| --- | --- | --- | --- | --- | --- | --- | --- | --- | --- | --- |
| Agarwal 2017^20^ | AML | Y | N (PubMed only) | U | U | N | Y | N | N |  |
| Moriwaki 2016^56^ | Biliary tract | Y | Y | U | Y | N | Y | Y | Y |  |
| Bruzzi 2005^23^ | Breast | Y | Y | U | Y | N | Y | N | Y |  |
| Burzykowski 2008^24^ | Breast | Y | N | U | U | N | Y | N | Y |  |
| Hackshaw 2005^36^ | Breast | Y | N (Medline only) | U | U | N | Y | Y | N |  |
| Liu 2016^51^ | Breast | Y | Y | Y | Y | N | Y | Y | Y |  |
| Petrelli 2014^64^ | Breast | Y | Y | U | U | N | Y | N | Y |  |
| Buyse 2000^25^ | Colorectal | Y | U | U | Y | N | Y | N | Y |  |
| Ciani 2015^26^ Elia 2018^33^ | Colorectal | Y | Y | Y | Y | Y | Y | Y | Y |  |
| Colloca 2016b^29^ | Colorectal | Y | Y | U | U | N | Y | N | N |  |
| Giessen 2015^35^ | Colorectal | Y | Y | U | U | N | Y | N | Y |  |
| Cremolini 2017^31^ | Colorectal | Y | Y | U | Y | N | Y | Y | N |  |
| Johnson 2006^46^ | Colorectal  Lung (NSCLC) | Y | Y | N | Y | Y | Y | N | N |  |
| Louvet 2001^52^ | Colorectal | Y | U | U | U | N | Y | N | N |  |
| Sidhu 2013^73^ | Colorectal | Y | Y | U | U | N | Y | Y | Y |  |
| Tang 2007^75^ | Colorectal | Y | Y | U | U | N | Y | N | Y |  |
| Tsujino 2010^77^ | Colorectal  Lung (NSCLC)  Various tumours | Y | N (PubMed only) | U | Y | N | Y | N | N |  |
| Ichikawa 2006^42^ | Gastric | Y | Y | U | U | N | Y | Y | N |  |
| Shitara 2014^70^ | Gastric | Y | Y | U | U | N | Y | N | Y |  |
| Pang 2018^61^ | Gastroesophageal | Y | N (search terms NR) | U | Y | Y | Y | N | N |  |
| Han 2014^38^ | Glioblastoma | Y | Y | U | U | N | Y | N | Y |  |
| Blumenthal 2017^22^ | Lung (NSCLC) | Y | N (trials submitted to FDA rather than search) | U | U | N | Y | N | Y |  |
| Blumenthal 2015^21^ | Lung (NSCLC) | Y | N (FDA trials not search) | U | U | N | Y | Y | Y |  |
| Hashim 2018^39^ | Lung (NSCLC) | Y | Y | Y | Y | Y | Y | Y | Y |  |
| Hotta 2015^40^ | Lung (NSCLC) | Y | Y | U | Y | N | Y | Y | N |  |
| Ito 2019^45^ | Lung (NSCLC) | Y | Y | Y | U | N | Y | Y | N |  |
| Li 2019^49^ | Lung (NSCLC) | Y | N (search terms NR) | U | Y | Y | Y | N | N |  |
| Li 2012^50^ | Lung (NSCLC) | Y | Y | Y | Y | N | Y | N | N |  |
| Nakashima 2016^58^ | Lung (NSCLC) | Y | Y | Y | Y | Y | Y | N | N |  |
| Ritchie 2018^65^ | Lung (NSCLC)  Various tumours | Y | Y | U | Y | Y | Y | N | Y |  |
| Roviello 2017^67^ | Lung (NSCLC)  Various tumours | Y | Y | Y | Y | N | Y | Y (for various) | Y |  |
| Sekine 1999^68^ | Lung (NSCLC) | Y | Y | U | U | N | Y | N | N |  |
| Shukuya 2016^71^ | Lung (NSCLC) | Y | Y | U | Y | N | Y | Y | N |  |
| Tsujino 2009^76^ | Lung (NSCLC) | Y | Y | U | U | N | N (slope only) | N | N |  |
| Vidaurre 2009^78^ | Lung (NSCLC)  Various | Y | N (trials in 5 journals rather than search) | U | Y | N | Y | Y (for various) | N |  |
| Foster 2011^34^ | Lung (SCLC) | Y | N (trials by 1 group rather than search) | U | U | N | Y | N | N |  |
| Hotta 2009^41^ | Lung (SCLC) | Y | Y | U | Y | N | Y | Y | N |  |
| Nickolich 2014^59^ | Lung (SCLC) | Y | N (trials in 1 journal rather than search) | U | Y | N | Y | Y | N |  |
| Mangal 2018^55^ (myeloma) | Multiple myeloma | Y | Y | U | U | N | Y | N | N |  |
| Imaoka 2019^44^ | Neuroendocrine | Y | Y | Y | U | N | Y | Y | Y |  |
| Imaoka 2017^43^ | Neuroendocrine | Y | Y | Y | U | N | Y | N | Y |  |
| Lee 2011^48^ | NHL (aggressive)  NHL (indolent) | Y | Y | U | U | N | Y | N | Y |  |
| Mangal 2018^54^ (NHL) | NHL | Y | Y | U | U | N | Y | N | N |  |
| Shi 2017^69^ | NHL (follicular) | Y | Y | U | U | N | Y | Y | Y |  |
| Zhu 2017^81^ | NHL (follicular)  NHL (mantle cell) | Y | Y | U | U | N | Y | N | Y |  |
| Colloca & Venturino 2017^27^ | Ovarian | Y | Y | Y | U | N | Y | Y | N |  |
| Rose 2010^66^ | Ovarian | Y | N (trials by 1 group rather than search) | N | U | N | Y | N | N |  |
| Siddiqui 2017^72^ | Ovarian | Y | Y | U | Y | N | Y | N | N |  |
| Colloca 2016a^28^ | Pancreatic | Y | N (PubMed only) | Y | U | N | Y | Y | N |  |
| Hamada 2016^37^ | Pancreatic | Y | Y | Y | Y | N | Y | N | Y |  |
| Makris 2017^53^ | Pancreatic (adenocarcinoma) | Y | N (search terms NR) | Y | Y | N | Y | N | Y |  |
| Colloca 2016c^30^ | Prostate | Y | N (PubMed only) | Y | Y | N | Y | Y | N |  |
| Abdel-Rahman 2018^18^ | Renal cell  Urothelial | Y | Y | U | U | N | Y | N | N |  |
| Delea 2012^32^ | Renal cell | Y | N (search terms NR) | Y | U | N | Y | N | N |  |
| Petrelli 2013^63^ | Renal cell | Y | Y | U | Y | N | Y | N | N |  |
| Tanaka 2019^74^ | Soft tissue sarcoma | Y | Y | U | Y | N | Y | N | Y |  |
| Zer 2016^80^ | Soft tissue sarcoma | Y | Y | U | Y | Y | Y | N | N |  |
| Penel 2014^62^ | Unknown primary | Y | N (Medline only; search terms NR) | U | U | N | Y | N | N |  |
| Agarwal 2014^19^ | Urothelial | Y | N (search methods NR) | U | U | N | Y | Y | N |  |
| Kaufman 2018^47^ | Various tumours | Y | Y | Y | Y | N | Y | Y | N |  |
| Mushti 2018^57^ | Various tumours | Y | N (FDA trials not search) | U | U | N | Y | N | N |  |
| Nie 2019^60^ | Various tumours | Y | Y | Y | Y | N | Y | N | N |  |
| Wilkerson+Fojo 2009^79^ | Various tumours | Y | N (search methods NR) | U | Y | N | Y | N | N |  |
| AML, acute myeloid leukaemia; N, No; NHL, non-Hodgkin’s lymphoma; NR, not reported; NSCLC, non-small cell lung cancer; SCLC, small cell lung cancer; U, Unclear; Y; Yes. | | | | | | | | | | |

Supplementary Table 7: Absolute correlation and regression results per study

| **Ref** | **SO** | **FO** | **Cancer** | **Line Sub-groups** | **Treatment** | **N stds** | **N pts** | **Absolute correlation methods** | **Correlation coefficient (95% CI), p-value** | **Absolute regression Methods** | **Regression R2 (95% CI), p-value** | **Linear regression equation** |
| --- | --- | --- | --- | --- | --- | --- | --- | --- | --- | --- | --- | --- |
| **ORR vs. PFS (or TTP)** | | | | | | | | | | | | |
| Louvet 2001^52^ | ORR | PFS | Colorectal | 1st | Various | 29 | 13498 | Spearman (ORR vs. med PFS) | rs=0.66, p<0.0001 | LR (ORR vs. med PFS) |  | PFS = 3.2 + 0.1 * ORR |
| Ichikawa 2006^42^ | ORR | TTP | Gastric | 1st | Chemo (any) | 12* | 2144 | Spearman, wtd O(RR vs. med TTP) | rs=0.49, p<0.0001 | WLR (ORR vs. med TTP) |  | TTP = 1.73 + 0.09 * ORR |
| Ichikawa 2006^42^ | ORR | TTP | Gastric | 1st | Chemo (novel) | 8* | 1077 | Spearman, wtd (ORR vs. med TTP) | rs=0.41, p=0.018 |  |  |  |
| Ichikawa 2006^42^ | ORR | TTP | Gastric | 1st | Chemo (non-novel) | 7* | 1067 | Spearman, wtd (ORR vs. med TTP) | rs=0.56, p=0.0053 |  |  |  |
| Ito 2019^45^ | ORR | PFS | Lung (NSCLC) | Various | Immune checkpoint inhibitors (PD-(L)1) | 6 | 3752^†^ | a) Pearson, wtd b) Spearman, wtd (ORR vs. med PFS) | a) r=0.55, p<0.0001 b) rs=0.33, p<0.0001 | WLR R2 (ORR vs. med PFS) | R2=0.30, p=0.206 |  |
| Ito 2019^45^ | ORR | PFS | Lung (NSCLC) | - Various - High PD-L1 expression | Immune checkpoint inhibitors (PD-(L)1) | 7 | 1381 | a) Pearson, wtd b) Spearman, wtd (ORR vs. med PFS) | a) r=0.90, p<0.0001 b) rs=0.48, p<0.0001 | WLR R2 (ORR vs. med PFS) | R2=0.81, p=0.006 |  |
| Ritchie 2018^65^ | ORR | PFS | Lung (NSCLC) | All | Immune checkpoint inhibitors (PD-(L)1 or CTLA4) | 8 | NR | Correlation (NR) (ORR vs. 6mo PFS) | r=0.85 (0.63 to 1.06), p=NR |  |  |  |
| Tsujino 2009^76^ | ORR | PFS | Lung (NSCLC) | NR | Targeted | 18* | 3790* |  |  | LR (ORR vs. med PFS) | R2=NR, p=0.001 | Slope 0.072 |
| Vidaurre 2009^78^ | ORR | PFS | Lung (NSCLC) | NR | Chemo or targeted | 35 | NR |  |  | Regression (NR) (ORR vs. med PFS) | R2=0.75, p<0.0001 |  |
| Nickolich 2014^59^ | ORR | PFS | Lung (SCLC) | - 1st + 2nd + maintenance - Limited or extensive | Various | 66^†^ | 8471^†^ | Pearson (ORR vs. med PFS) | r=0.73, p<0.0001 |  |  |  |
| Nickolich 2014^59^ | ORR | PFS | Lung (SCLC) | - 1st + 2nd + maintenance - Limited disease | Various | 66^†^ | 8471^†^ | Pearson (ORR vs. med PFS) | r=0.02, p=0.978 |  |  |  |
| Nickolich 2014^59^ | ORR | PFS | Lung (SCLC) | - 1st + 2nd + maintenance - Extensive disease | Various | 66^†^ | 8471^†^ | Pearson (ORR vs. med PFS) | r=0.51, p=0.013 |  |  |  |
| Mangal 2018^55^ (myeloma) | ORR | PFS | Multiple myeloma | 2nd + | Various | 79^†^ | 13322^†^ |  |  | WLR adj R2 (logit ORR vs. log med PFS) | Adj R2=0.50, p=NR |  |
| Imaoka 2019^44^ | ORR | PFS | Neuroendocrine | Various | Systemic | 22 | 1310 | Pearson (ORR vs. med PFS) | r=0.37 (-0.05 to 0.80), p=0.085 |  |  |  |
| Imaoka 2019^44^ | ORR | PFS | Neuroendocrine | - Various - Published 1996-2010 | Systemic | 6* | NR | Pearson (ORR vs. med PFS) | r= -0.08 (-0.76 to 0.60), p=0.824 |  |  |  |
| Imaoka 2019^44^ | ORR | PFS | Neuroendocrine | - Various - Published 2011-2016 | Systemic | 16* | NR | Pearson (ORR vs. med PFS) | r=0.43 (-0.07 to 0.93), p=0.095 |  |  |  |
| Imaoka 2019^44^ | ORR | PFS | Neuroendocrine | Various | Cytotoxic | 9 arms | NR | Pearson (ORR vs. med PFS) | r=0.63 (0.03 to 1.22), p=0.041 |  |  |  |
| Imaoka 2019^44^ | ORR | PFS | Neuroendocrine | Various | Non-cytotoxic | 18 arms | NR | Pearson (ORR vs. med PFS) | r=0.18 (-0.27 to 0.62), p=0.432 |  |  |  |
| Imaoka 2019^44^ | ORR | PFS | Neuroendocrine | Various | Targeted | 19 arms | NR | Pearson (ORR vs. med PFS) | r=0.42 (-0.06 to 0.90), p=0.086 |  |  |  |
| Imaoka 2019^44^ | ORR | PFS | Neuroendocrine | Various | Non-targeted | 8 arms | NR | Pearson (ORR vs. med PFS) | r= -0.72 (-1.09 to -0.35), p<0.001 |  |  |  |
| Mangal 2018^54^ (NHL) | ORR | PFS | NHL | Various | Various | 73 | 6071 |  |  | LR adj R2 (logit ORR vs. log med PFS) | Adj R2=0.70, p=NR | log (med PFS) = 1.97 + 0.414 * logit (ORR) |
| Rose 2010^66^ | ORR | PFS | Ovarian | 2nd | Various | 11 | 407 | a) Pearson b) Kendall Tau-b (ORR vs. med PFS) | a) r=0.62, p=0.044  b) r=0.48, p=0.042 |  |  |  |
| Siddiqui 2017^72^ | ORR | PFS | Ovarian | 2nd + | Chemo | 39^†^ | 9223^†^ | a) Pearson, wtd (ORR vs. med PFS) b) Pearson, unwtd (ORR vs. med PFS) | a) r=0.85, p<0.001 b) 0.76, p<0.001 | WLR R2 (ORR vs. med PFS): a) unadj b) adj | a) R2=0.72, p=NR b) adj R2=0.72, p=NR | med PFS = 2.59 + 0.12 * ORR |
| Petrelli 2013^63^ | ORR | PFS | Renal cell | 1st | Targeted | 6^†^ | 3188^†^ | Spearman, wtd (ORR vs. med PFS) | rs=0.96, p<0.0001 |  |  |  |
| Penel 2014^62^ | ORR | PFS | Unknown primary | NR | NR | 38^†^ | NR | Pearson via WLR (ORR v. med PFS) | r=0.54, p<0.0001 |  |  |  |
| Ritchie 2018^65^ | ORR | PFS | Various solid tumours | All | Immune checkpoint inhibitors (PD-(L)1 or CTLA4) | 20^†^ | 10828^†^ | Correlation (NR) (ORR vs. 6mo PFS) | r=0.37 (0.06 to 0.95), p=NR |  |  |  |
| Vidaurre 2009^78^ | ORR | PFS | Various | NR | Chemo | 85 | 3982* |  |  | Regression (NR) (ORR vs. med PFS) | R2=0.53, p<0.0001 |  |
| Vidaurre 2009^78^ | ORR | PFS | Various | NR | Targeted | 58 | 2992* |  |  | Regression (NR) (ORR vs. med PFS) | R2=0.61, p<0.0001 |  |
| Vidaurre 2009^78^ | ORR | PFS | Various | NR | Chemo or targeted | 143^†^ | 6974^†^ |  |  | Regression (NR) (ORR vs. med PFS) | R2=0.56, p<0.0001 |  |
| **ORR vs. OS** | | | | | | | | | | | | |
| Agarwal 2017^20^ | ORR | OS | Acute myeloid leukemia | 1st | Systemic | 20^†^ | NR |  |  | WLR adj R2 (logit ORR vs. log med OS) | Adj R2=0.45, p=NR |  |
| Liu 2016^51^ | ORR | OS | Breast | 2nd + 3rd | Chemo | 24 | 8617 | Spearman (ORR vs. med OS) | rs=0.54 (0.29 to 0.72), p<0.0001 |  |  |  |
| Liu 2016^51^ | ORR | OS | Breast | - 2nd + 3rd - Previous anthracycline/taxanes | Chemo | 15* | NR | Spearman (ORR vs. med OS) | rs=0.62 (0.32 to 0.84), p=NR |  |  |  |
| Liu 2016^51^ | ORR | OS | Breast | - 2nd + 3rd - Previous trastuzumab/bevacizumab | Chemo | 5* | NR | Spearman (ORR vs. med OS) | rs=0.78 (0.19 to 1.0), p=NR |  |  |  |
| Liu 2016^51^ | ORR | OS | Breast | 2nd + 3rd | Chemo (taxanes) | 21* | NR | Spearman (ORR vs. med OS) | rs=0.49 (-0.19 to 0.92), p=NR |  |  |  |
| Liu 2016^51^ | ORR | OS | Breast | 2nd + 3rd | Chemo (antimetabolites) | 22* | NR | Spearman (ORR vs. med OS) | rs=-0.10, p=NR |  |  |  |
| Liu 2016^51^ | ORR | OS | Breast | - 2nd + 3rd - HER2-pos | Chemo | 5* | NR | Spearman (ORR vs. med OS) | rs=0.96 (0.80 to 1.00), p=NR |  |  |  |
| Liu 2016^51^ | ORR | OS | Breast | - 2nd + 3rd - HER2-neg | Chemo | 3* | NR | Spearman (ORR vs. med OS) | rs=1.00, p=NR |  |  |  |
| Petrelli 2014^64^ | ORR | OS | Breast | 1st | Targeted + chemo | 20^†^ | 10138^†^ | Spearman, wtd (ORR vs. med OS) | rs=0.61 (0.59 to 0.63), p=NR |  |  |  |
| Giessen 2015^35^ | ORR | OS | Colorectal | 2nd | Chemo | 22 | 10509 | Pearson, wtd (log odds ORR vs. log med OS) | r=0.58 (0.38 to 0.72), p=0.003 |  |  |  |
| Louvet 2001^52^ | ORR | OS | Colorectal | 1st | Various | 28* | 13284* | Spearman (ORR vs. med OS) | rs=0.41, p=0.0009 | LR (ORR vs. med OS) |  | OS = 10.45 + 0.088 * ORR |
| Tang 2007^75^ | ORR | OS | Colorectal | 1st | Chemo | 39 | 18668 | Spearman (ORR vs. med OS) | rs=0.59 (0.42 to 0.72), p<0.000001 |  |  |  |
| Ichikawa 2006^42^ | ORR | OS | Gastric | 1st | Chemo (any) | 25 | 4593 | Spearman, wtd (ORR vs. med OS) | rs=0.45, p<0.0001 | WLR (ORR vs. med OS) |  | OS = 5.89 + 0.08 * ORR |
| Ichikawa 2006^42^ | ORR | OS | Gastric | 1st | Chemo (novel) | 11* | 1170 | Spearman, wtd (ORR vs. med OS) | rs=0.18, p=0.12 |  |  |  |
| Ichikawa 2006^42^ | ORR | OS | Gastric | 1st | Chemo (non-novel) | 20* | 3423 | Spearman, wtd (ORR vs. med OS) | rs=0.47, p<0.0001 |  |  |  |
| Shitara 2014^70^ | ORR | OS | Gastric | 2nd + 3rd | Chemo | 64 | 4286 | Spearman (ORR vs. med OS) | rs=0.38 (0.16 to 0.6), p=NR |  |  |  |
| Pang 2018^61^ | ORR | OS | Gastroesophageal | 1st + 2nd | Targeted | 18 | 7892 | Correlation (NR) (ORR vs. med OS) | r=0.86, p<0.0001 |  |  |  |
| Han 2014^38^ | ORR | OS | Glioblastoma | Various | Various | 91^†^ | 7125^†^ |  |  | WLR R2 (ORR vs. med OS) | R2=0.22 (0.04 to 0.42), p=NR |  |
| Ito 2019^45^ | ORR | OS | Lung (NSCLC) | Various | Immune checkpoint inhibitors (PD-(L)1) | 6 | 3752^†^ | a) Pearson, wtd b) Spearman, wtd (ORR vs. med OS) | a) r= -0.02, p=0.4564 b) rs= -0.14, p<0.0001 |  |  |  |
| Ito 2019^45^ | ORR | OS | Lung (NSCLC) | - Various - High PD-L1 expression | Immune checkpoint inhibitors (PD-(L)1) | 7 | 1381 | a) Pearson, wtd b) Spearman, wtd (ORR vs. med OS) | a) r=0.92, p<0.0001 b) rs=0.77, p<0.0001 | WLR R2 (ORR vs. med OS) | R2=0.84, p=0.004 |  |
| Li 2019^49^ | ORR | OS | Lung (NSCLC) | 1st + 2nd | Immune checkpoint inhibitors | 5^†^ | 4803^†^ | Pearson (ORR vs. med OS) | r=0.52, p=0.28 | LR (ORR vs. med OS) | R2=0.27, p=NR |  |
| Li 2012^50^ | ORR | OS | Lung (NSCLC) | 1st + 2nd | Targeted | 60 | 9903 |  |  | WLSR R2 (ORR vs. med OS) | R2=0.83, p<0.000001 |  |
| Ritchie 2018^65^ | ORR | OS | Lung (NSCLC) | All | Immune checkpoint inhibitors (PD-(L)1 or CTLA4) | 8 | NR | Correlation (NR) (ORR vs. 12mo OS) | r=0.66 (0.17 to 1.08), p=NR |  |  |  |
| Sekine 1999^68^ | ORR | OS | Lung (NSCLC) | Various | Chemo | 42 | 1935 | Pearson (ORR vs. med OS) | r=0.62, p<0.001 |  |  |  |
| Shukuya 2016^71^ | ORR | OS | Lung (NSCLC) | All | Immune checkpoint inhibitors (PD-(L)1) | 10^†^ | NR | Spearman, wtd (ORR vs. med OS) | rs=0.45, p=0.141 |  |  |  |
| Shukuya 2016^71^ | ORR | OS | Lung (NSCLC) | All | Chemo (docetaxel) | 22^†^ | NR | Spearman, wtd (ORR vs. med OS) | rs=0.41, p=0.053 |  |  |  |
| Tsujino 2009^76^ | ORR | OS | Lung (NSCLC) | NR | Targeted | 28 | 6171 |  |  | LR (ORR vs. med OS) | R2=NR, p<0.0001 | Slope 0.258 |
| Vidaurre 2009^78^ | ORR | OS | Lung (NSCLC) | NR | Chemo or targeted | 35 | NR |  |  | Regression (NR) (ORR vs. med OS) | R2=0.28, p=0.0024 |  |
| Nickolich 2014^59^ | ORR | OS | Lung (SCLC) | - 1st + 2nd + maintenance - Limited or extensive | Various | 66^†^ | 8471^†^ | Pearson (ORR vs. med OS) | r=0.66, p<0.0001 |  |  |  |
| Nickolich 2014^59^ | ORR | OS | Lung (SCLC) | - 1st + 2nd + maintenance - Limited disease | Various | 66^†^ | 8471^†^ | Pearson (ORR vs. med OS) | r=0.40, p=0.193 |  |  |  |
| Nickolich 2014^59^ | ORR | OS | Lung (SCLC) | - 1st + 2nd + maintenance - Extensive disease | Various | 66^†^ | 8471^†^ | Pearson (ORR vs. med OS) | r=0.44, p=0.012 |  |  |  |
| Imaoka 2017^43^ | ORR | OS | Neuroendocrine | Various | Systemic | 20 | 2530 | Spearman (ORR vs. med OS) | rs= -0.26 (-0.64 to 0.11), p=0.164 |  |  |  |
| Rose 2010^66^ | ORR | OS | Ovarian | 2nd | Various | 11 | 407 | a) Pearson b) Kendall Tau-b (ORR vs. med OS) | a) r=0.56, p=0.071  b) r=0.40, p=0.086 |  |  |  |
| Siddiqui 2017^72^ | ORR | OS | Ovarian | 2nd + | Chemo | 31^†^ | 9223^†^ | a) Pearson, wtd (ORR vs. med OS) b) Pearson, unwtd (ORR vs. med OS) | a) r=0.82, p<0.001 b) 0.71, p<0.001 | WLR R2 (ORR vs. med OS): a) unadj b) adj | a) R2=0.67, p=NR b) adj R2=0.66, p=NR | med OS = 9.48 + 0.28 * ORR |
| Hamada 2016^37^ | ORR | OS | Pancreatic | 1st | Chemo | 47 | 15906^†^ | Spearman (ORR vs. med OS) | rs=0.39 (0.20 to 0.55), p<0.001 |  |  |  |
| Abdel-Rahman 2018^18^ | ORR | OS | Renal cell | Various | Immune checkpoint inhibitors (PD-(L)1) | 4 | 1093 | Pearson (ORR vs. med OS) | r= -0.40, p=0.436 |  |  |  |
| Petrelli 2013^63^ | ORR | OS | Renal cell | 1st | Targeted | 6^†^ | 3188^†^ | Spearman, wtd (ORR vs. med OS) | rs=0.96, p<0.0001 |  |  |  |
| Penel 2014^62^ | ORR | OS | Unknown primary | NR | NR | 38^†^ | NR | Pearson via WLR (ORR v. med OS) | r=0.54, p<0.0001 |  |  |  |
| Abdel-Rahman 2018^18^ | ORR | OS | Urothelial | Various | Immune checkpoint inhibitors (PD-(L)1) | 9 | 1699 | Pearson (ORR vs. med OS) | r= -0.12, p=0.758 |  |  |  |
| Agarwal 2014^19^ | ORR | OS | Urothelial | 2nd | Chemo or biologic | 10 | 560 | Pearson (ORR vs. 12mo OS) | r=0.37, p=0.30 | WLR R2 (ORR vs. 12mo OS): a) unadj b) adj (RE) | a) R2=0.26, p=NR b) Adj R2=0.16, p=0.1359 |  |
| Agarwal 2014^19^ | ORR | OS | Urothelial | - 2nd - Operable | Chemo | NR | 214^†^ | Pearson (ORR vs. 12mo OS) | r=0.78, p=NR | WLR adj R2 (ORR vs. 12mo OS) | Adj R2=0.54, p=NR |  |
| Agarwal 2014^19^ | ORR | OS | Urothelial | - 2nd - Metastatic | Chemo | NR | 391^†^ | Pearson (ORR vs. 12mo OS) | r= -0.018, p=NR | WLR adj R2 (ORR vs. 12mo OS) | Adj R2= -0.13, p=NR |  |
| Nie 2019^60^ | ORR | OS | Various solid tumours | Various | Immune checkpoint inhibitors (PD-(L)1) | 43^†^ | 15088^†^ |  |  | Squared Spearman (ORR vs. med OS) | r2s=0.29, p<0.001 |  |
| Ritchie 2018^65^ | ORR | OS | Various solid tumours | All | Immune checkpoint inhibitors (PD-(L)1 or CTLA4) | 20^†^ | 10828^†^ | Correlation (NR) (ORR vs. 12mo OS) | r=0.08 (-0.17 to 0.70), p=NR |  |  |  |
| Vidaurre 2009^78^ | ORR | OS | Various | NR | Chemo | 85 | 3982* |  |  | Regression (NR) (ORR vs. med OS) | R2=0.35, p<0.0001 |  |
| Vidaurre 2009^78^ | ORR | OS | Various | NR | Targeted | 58 | 2992* |  |  | Regression (NR) (ORR vs. med OS) | R2=0.45, p<0.0001 |  |
| Vidaurre 2009^78^ | ORR | OS | Various | NR | Chemo or targeted | 143^†^ | 6794^†^ |  |  | Regression (NR) (ORR vs. med OS) | R2=0.33, p<0.0001 |  |
| **CR vs. PFS** | | | | | | | | | | | | |
| Nickolich 2014^59^ | CR | PFS | Lung (SCLC) | - 1st + 2nd + maintenance - Limited or extensive | Various | 66^†^ | 8471^†^ | Pearson (CR vs. med PFS) | r=0.71, p<0.0001 |  |  |  |
| Nickolich 2014^59^ | CR | PFS | Lung (SCLC) | - 1st + 2nd + maintenance - Limited disease | Various | 66^†^ | 8471^†^ | Pearson (CR vs. med PFS) | r=0.22, p=0.491 |  |  |  |
| Nickolich 2014^59^ | CR | PFS | Lung (SCLC) | - 1st + 2nd + maintenance - Extensive disease | Various | 66^†^ | 8471^†^ | Pearson (CR vs. med PFS) | r=0.35, p=0.116 |  |  |  |
| Mangal 2018^55^ (myeloma) | CR | PFS | Multiple myeloma | 2nd + | Various | 79^†^ | 13322^†^ |  |  | WLR adj R2 (logit CR vs. log med PFS) | Adj R2=0.47, p=NR |  |
| Mangal 2018^54^ (NHL) | CR | PFS | NHL | Various | Various | 73 | 6071 |  |  | LR adj R2 (logit CR vs. log med PFS) | Adj R2=0.57, p=NR | log (med PFS) = 2.38 + 0.340 * logit (CR) |
| Zhu 2017^81^ | CR | PFS | NHL (indolent; follicular) | NR | Chemo, immune or targeted | 13 | NR |  |  | WLR R2: a) CR vs. med PFS b) CR vs. 3-year PFS | a) R2=0.69 (0.22 to 0.89), p=NR  b) R2=0.44, p=NR | med PFS = 0.83 + 0.46 * CR |
| Zhu 2017^81^ | CR | PFS | NHL (mantle cell) | NR | Chemo, immune or targeted | NR | NR |  |  | WLR R2 (CR vs. med PFS) | R2=0.39, p=NR |  |
| **CR vs. OS** | | | | | | | | | | | | |
| Agarwal 2017^20^ | CR | OS | Acute myeloid leukemia | 1st | Systemic | 20^†^ | NR |  |  | WLR adj R2 (logit CR vs. log med OS) | Adj R2=0.48, p=NR |  |
| Pang 2018^61^ | CR | OS | Gastroesophageal | 1st + 2nd | Targeted | 18 | 7892 | Correlation (NR) (CR vs. med OS) | r=0.43, p=0.18 |  |  |  |
| Li 2019^49^ | CR | OS | Lung (NSCLC) | 1st + 2nd | Immune checkpoint inhibitors | 5* | 4103* | Pearson (CR vs. med OS) | r=0.19, p=0.75 | LR (CR vs. med OS) | R2=0.04, p=NR |  |
| Nickolich 2014^59^ | CR | OS | Lung (SCLC) | - 1st + 2nd + maintenance - Limited or extensive | Various | 66^†^ | 8471^†^ | Pearson (CR vs. med OS) | r=0.62, p<0.0001 |  |  |  |
| Nickolich 2014^59^ | CR | OS | Lung (SCLC) | - 1st + 2nd + maintenance - Limited disease | Various | 66^†^ | 8471^†^ | Pearson (CR vs. med OS) | r=-0.04, p=0.863 |  |  |  |
| Nickolich 2014^59^ | CR | OS | Lung (SCLC) | - 1st + 2nd + maintenance - Extensive disease | Various | 66^†^ | 8471^†^ | Pearson (CR vs. med OS) | r=0.19, p=0.295 |  |  |  |
| **PR (or VGPR or CR) vs. PFS** | | | | | | | | | | | | |
| Nickolich 2014^59^ | PR | PFS | Lung (SCLC) | - 1st + 2nd + maintenance - Limited or extensive | Various | 66^†^ | 8471^†^ | Pearson (PR vs. med PFS) | r=0.35, p=0.019 |  |  |  |
| Nickolich 2014^59^ | PR | PFS | Lung (SCLC) | - 1st + 2nd + maintenance - Limited disease | Various | 66^†^ | 8471^†^ | Pearson (PR vs. med PFS) | r=0.70, p=0.011 |  |  |  |
| Nickolich 2014^59^ | PR | PFS | Lung (SCLC) | - 1st + 2nd + maintenance - Extensive disease | Various | 66^†^ | 8471^†^ | Pearson (PR vs. med PFS) | r=0.49, p=0.035 |  |  |  |
| Mangal 2018^55^ (myeloma) | VGPR or CR | PFS | Multiple myeloma | 2nd + | Various | 79^†^ | 13322^†^ |  |  | WLR adj R2 (VGPR or CR vs. med PFS) | Adj R2=0.64, p=NR |  |
| **PR vs. OS** | | | | | | | | | | | | |
| Nickolich 2014^59^ | PR | OS | Lung (SCLC) | - 1st + 2nd + maintenance - Limited or extensive | Various | 66^†^ | 8471^†^ | Pearson (PR vs. med OS) | r=0.29, p=0.018 |  |  |  |
| Nickolich 2014^59^ | PR | OS | Lung (SCLC) | - 1st + 2nd + maintenance - Limited disease | Various | 66^†^ | 8471^†^ | Pearson (PR vs. med OS) | r=0.60, p=0.009 |  |  |  |
| Nickolich 2014^59^ | PR | OS | Lung (SCLC) | - 1st + 2nd + maintenance - Extensive disease | Various | 66^†^ | 8471^†^ | Pearson (PR vs. med OS) | r=0.66, p=0.0002 |  |  |  |
| *Calculated from reported data. ^†^Unclear for individual subgroups.  adj, adjusted; chemo, chemotherapy; CI, confidence interval; CR, complete response; FO, final outcome; HER2, human epidermal growth factor receptor 2; log, logarithm; LR, linear regression; med, median; mo, months; NHL, non-Hodgkin’s lymphoma; NR, not reported; NSCLC, non-small cell lung cancer; ORR, overall response rate (ORR=PR+CR); OS, overall survival; PFS, progression-free survival; PR, partial response; r, Pearson correlation; R2, regression coefficient of determination; r2s, squared Spearman rank correlation; rs, Spearman rank correlation; SCLC, small cell lung cancer; SO, surrogate outcome; TTP, time to progression; unwtd, unweighted; VGPR, very good partial response; wtd, weighted; WLR, weighted linear regression; WLSR, weighted least squares regression. | | | | | | | | | | | | |

Supplementary Table 8: Treatment effect correlation and regression results per study

| **Ref** | **SO** | **FO** | **Cancer** | **Line Sub-groups** | **Treatment** | **N stds** | **N pts** | **Treatment effect correlation methods** | **Correlation coefficient (95% CI), p-value** | **Treatment effect regression methods** | **Regression R2 (95% CI), p-value** | **Linear regression equation** | **STE** | **IQWiG** | **BSES2** |
| --- | --- | --- | --- | --- | --- | --- | --- | --- | --- | --- | --- | --- | --- | --- | --- |
| **ORR vs. PFS** | | | | | | | | | | | | | | | |
| Burzykowski 2008^24^ | ORR | PFS | Breast | 1st | Chemo | 11 | 3953 | Spearman via LR with Plackett copula (logOR ORR vs. logHR PFS) | rs=0.96 (0.73 to 1.19), p=NR | LR |  | logHR PFS = 0.10 + 0.50 * logOR ORR | NR | Medium+ | NE |
| Ciani 2015^26^ Elia 2018^33^ | ORR | PFS | Colorectal | All | Systemic | 33 | NR |  |  | LR: Adj R2 (logOR ORR vs. logHR PFS) | Adj R2=0.61 (0.27 to 0.87), p=NR | logHR PFS = -0.05 - 0.32 * logOR ORR | NR | Medium | NE |
| Ciani 2015^26^ Elia 2018^33^ | ORR | PFS | Colorectal | - All - No crossover | Systemic | 7 | NR |  |  | LR: Adj R2 (logOR ORR vs. logHR PFS) | Adj R2=0.63 (0.03 to 0.99), p=NR | logHR PFS = -0.05 - 0.31 * logOR ORR | NR | Medium | NE |
| Tsujino 2010^77^ | ORR | PFS | Colorectal | NR | Targeted | 7 | NR |  |  | LR (unwtd) R2 (diff ORR vs. HR PFS) | R2=0.65, p=0.029 | Slope -0.037 | NR | Medium | NE |
| Blumenthal 2017^22^ | ORR | PFS | Lung (NSCLC) | Various | Chemo, immune or targeted | 25 | 20013^†^ |  |  | WLR R2: a) OR ORR vs. HR PFS b) 6mo ratio ORR vs. HR PFS | a) R2=0.74 (0.55 to 0.88), p=NR b) R2=0.70 (0.50 to 0.84), p=NR |  | NR | Medium+ | NE |
| Blumenthal 2015^21^ | ORR | PFS | Lung (NSCLC) | Various | Chemo or targeted | 14 | 12567^†^ |  |  | WLR R2 (logOR ORR vs. logHR PFS) | R2=0.89 (0.80 to 0.98), p=NR |  | NR | Medium+ | NE |
| Blumenthal 2015^21^ | ORR | PFS | Lung (NSCLC) | Various | Chemo | 11 | 11701^†^ |  |  | WLR R2 (logOR ORR vs. logHR PFS) | R2=0.77 (0.58 to 0.96), p=NR |  | NR | Medium+ | NE |
| Ito 2019^45^ | ORR | PFS | Lung (NSCLC) | Various | Immune (PD-(L)1) | 6 | 3752^†^ | a) Pearson, wtd b) Spearman, wtd (OR ORR vs. HR PFS) | a) r= -0.87, p<0.0001 b) rs= -0.97, p<0.0001 | WLR R2 (OR ORR vs. HR PFS) | R2=0.76, p=0.011 |  | NR | Medium+ | Fair |
| Ito 2019^45^ | ORR | PFS | Lung (NSCLC) | - Various - High PD-L1 expression | Immune checkpoint inhibitors (PD-(L)1) | 7 | 1381 | a) Pearson, wtd b) Spearman, wtd (OR ORR vs. HR PFS) | a) r=0.67, p<0.0001 b) rs=0.56, p<0.0001 | WLR R2 (OR ORR vs. HR PFS) | R2=0.45, p=0.101 |  | NR | Low | Good |
| Ritchie 2018^65^ | ORR | PFS | Lung (NSCLC) | All | Immune checkpoint inhibitors (PD-(L)1 or CTLA4) | 8 | NR | Correlation (NR), wtd (OR ORR vs. HR PFS) | r=0.74 (0.38 to 1.08), p=NR |  |  |  | NR | Medium | Good |
| Roviello 2017^67^ | ORR | PFS | Lung (NSCLC) | Various | Immune checkpoint inhibitors | 7* | 3369* |  |  | WLR R2 (logOR ORR vs. logHR PFS) | R2=0.42 (0.003 to 0.85), p=0.06 |  | NR | Low | NE |
| Tsujino 2010^77^ | ORR | PFS | Lung (NSCLC) | NR | Targeted | 6 | NR |  |  | LR (unwtd) R2 (diff ORR vs. HR PFS) | R2=0.94, p=0.002 | Slope -0.015 | NR | Medium+ | NE |
| Colloca & Venturino 2017^27^ | ORR | PFS | Ovarian | 1st | Chemo | 29 | NR | Spearman (diff ORR vs. diff med PFS) | rs=0.64, p<0.001 | LR R2 (log RR ORR vs. log HR PFS) | R2=0.28, p=0.005 |  | NR | Low | NE |
| Colloca & Venturino 2017^27^ | ORR | PFS | Ovarian | - 1st - Published 1990-2002 | Chemo | 15 | NR | Spearman (diff ORR vs. diff med PFS) | rs=0.64, p=0.018 | LR R2 (log RR ORR vs. log HR PFS) | R2=0.32, p=0.046 |  | NR | Low | NE |
| Colloca & Venturino 2017^27^ | ORR | PFS | Ovarian | - 1st - Published 2003-2016 | Chemo | 16 | NR | Spearman (diff ORR vs. diff med PFS) | rs=0.58, p=0.019 | LR R2 (log RR ORR vs. log HR PFS) | R2=0.53, p=0.003 |  | NR | Medium | NE |
| Siddiqui 2017^72^ | ORR | PFS | Ovarian | 2nd + | Chemo | 39^†^ | 9223^†^ | Pearson, wtd (OR ORR vs. HR PFS) | r=0.42, p=NR |  |  |  | NR | Low | Poor |
| Colloca 2016a^28^ | ORR | PFS | Pancreatic | 1st | Gemcitabine + chemo or targeted | 33* | NR | Spearman (diff ORR vs. diff med PFS) | rs=0.34, p=NR |  |  |  | NR | Low | NE |
| Colloca 2016a^28^ | ORR | PFS | Pancreatic | 1st | Gemcitabine + targeted | 14* | NR | Spearman (diff ORR vs. diff med PFS) | rs=0.25, p=NR |  |  |  | NR | Low | NE |
| Ritchie 2018^65^ | ORR | PFS | Various solid tumours | All | Immune checkpoint inhibitors (PD-(L)1 or CTLA4) | 20^†^ | 10828^†^ | Correlation (NR), wtd (OR ORR vs. HR PFS) | r=0.63 (0.35 to 0.89), p=NR |  |  |  | NR | Medium | Poor |
| Roviello 2017^67^ | ORR | PFS | Various solid tumours | Various | Immune checkpoint inhibitors | 17^†^ | 8994^†^ |  |  | WLR R2 (logOR ORR vs. logHR PFS) | R2=0.32 (0.02 to 0.76), p=0.01 | logHR PFS = -0.1281 - 0.2384 * logOR ORR | NR | Low | NE |
| Roviello 2017^67^ | ORR | PFS | Various solid tumours | Various | Immune checkpoint inhibitors (CTLA-4) | 17^†^ | 8994^†^ |  |  | WLR R2 (logOR ORR vs. logHR PFS) | R2=0.67 (0.02 to 1.00), p=0.05 |  | NR | Medium | NE |
| Roviello 2017^67^ | ORR | PFS | Various solid tumours | Various | Immune checkpoint inhibitors (PD-(L)1) | 17^†^ | 8994^†^ |  |  | WLR R2 (logOR ORR vs. logHR PFS) | R2=0.25 (0.02 to 1.00), p=0.08 |  | NR | Low | NE |
| Tsujino 2010^77^ | ORR | PFS | Various solid tumours | NR | Targeted | 17 | NR |  |  | LR (unwtd) R2 (diff ORR vs. HR PFS) | R2=0.50, p=0.001 | Slope -0.022 | 15% | Medium | NE |
| Wilkerson+Fojo 2009^79^ | ORR | PFS | Various solid tumours | NR | NR | 66^†^ | NR |  |  | LR (unwtd R2): a) diff ORR vs. HR PFS b) diff ORR vs. diff med PFS | a) R2=0.45, p<0.0001 b) R2=0.62, p<0.0001 |  | NR | Medium | NE |
| **ORR vs. OS** | | | | | | | | | | | | | | | |
| Moriwaki 2016^56^ | ORR | OS | Biliary tract | 1st | Chemo | 17^†^ | 2040 |  |  | WLR R2 (ratio ORR vs. log ratio med OS) | R2=0.29 (0.01 to 0.65), p=0.021 | log ratio med OS = 0.013 + 0.282 * ratio ORR | NR | Low | NE |
| Moriwaki 2016^56^ | ORR | OS | Biliary tract | 1st | Chemo (gemcitabine) | 14^†^ | 1880 |  |  | WLR R2 (ratio ORR vs. log ratio med OS) | R2=0.39 (0.02 to 0.75), p=0.013 | log ratio med OS = 0.020 + 0.268 * ratio ORR | NR | Low | NE |
| Moriwaki 2016^56^ | ORR | OS | Biliary tract | 1st | Targeted | 6^†^ | 953 |  |  | WLR R2 (ratio ORR vs. log ratio med OS) | R2=0.43 (0.03 to 0.89), p=0.090 | log ratio med OS = 0.119 + 0.155 * ratio ORR | NR | Low | NE |
| Bruzzi 2005^23^ | ORR | OS | Breast | All | Chemo | 10 | 2126 |  |  | WLR R2: a) logOR ORR vs. logHR OS b) diff ORR vs. diff med OS | a) R2=0.10 (0.00 to 0.43), p=NR b) R2=0.20 (0 to 0.65), p=NR |  | NR | Low | NE |
| Burzykowski 2008^24^ | ORR | OS | Breast | 1st | Chemo | 11 | 3953 | Spearman via LR with Plackett copula (logOR ORR vs. logHR OS) | rs=0.57 (-0.31 to 1.44), p=NR |  |  |  | NR | Medium | NE |
| Hackshaw 2005^36^ | ORR | OS | Breast | 1st | Chemo | 42* | 9163 |  |  | WLR R2 (logOR ORR vs. logHR OS) | R2=0.34, p<0.0001 | logHR OS = -0.0081 + 0.28 * logOR ORR  Slope 0.28 | NR | Low | NE |
| Hackshaw 2005^36^ | ORR | OS | Breast | - 1st - Recruited pre-1990 | Chemo | 26* | 5244* |  |  | WLR R2 (logOR ORR vs. logHR OS) | R2=0.26, p=0.004 | Slope 0.28 | NR | Low | NE |
| Hackshaw 2005^36^ | ORR | OS | Breast | - 1st - Recruited 1990 or after | Chemo | 16* | 3919* |  |  | WLR R2 (logOR ORR vs. logHR OS) | R2=0.41, p=0.005 | Slope 0.24 | NR | Low | NE |
| Buyse 2000^25^ | ORR | OS | Colorectal | 1st | Chemo | 25 | 3791 |  |  | WLR R2 (logOR ORR vs. logHR OS) | R2=0.38 (0.09 to 0.68), p=NR |  | NR | Low | NE |
| Ciani 2015^26^ Elia 2018^33^ | ORR | OS | Colorectal | All | Systemic | 32 | NR | Spearman (logOR ORR vs. logOR OS) | rs=0.53, p<0.01 | a) WLSR R2 (logOR ORR vs. logOR OS) (timepoint NR) b) Adj R2 (logOR ORR vs. logHR OS) | a) R2=0.06 (0.01 to 0.29), p=NR b) Adj R2=0.33 (0.00 to 0.91), p=NR | logHR OS = -0.03 - 0.05 * logOR ORR | 0.28 | Low | NE |
| Ciani 2015^26^ Elia 2018^33^ | ORR | OS | Colorectal | - All - No crossover | Systemic | 7 | NR |  |  | LR: Adj R2 (logOR ORR vs. logHR OS) | Adj R2=0.40 (0.00 to 0.96), p=NR | logHR OS = -0.04 - 0.10 * logOR ORR | NR | Low | NE |
| Colloca 2016b^29^ | ORR | OS | Colorectal | 1st | Bevacizumab + chemo | 11 | NR | Spearman (diff ORR vs. diff med OS) | rs=0.82, p<0.001 | LR R2 (diff ORR vs. diff med OS) | R2=0.58, p=0.002 |  | NR | Medium | NE |
| Cremolini 2017^31^ | ORR | OS | Colorectal | 2nd | Targeted | 20* | 7571 | Pearson (via WLR): a) rr ORR vs. HR OS b) diff ORR vs. diff med OS | a) r=0.17, p=0.476 b) r=0.35, p=0.092 | WLR R2: a) rr ORR vs. HR OS b) diff ORR vs. diff med OS | b) R2=0.03, p=0.476 b) R2=0.12, p=0.092 | a) Slope -0.029 b) Slope 0.071 | NR | Low | NE |
| Cremolini 2017^31^ | ORR | OS | Colorectal | 2nd | Targeted, anti-angiogenic | 13* | NR | Pearson (via WLR): a) rr ORR vs. HR OS b) diff ORR vs. diff med OS | a) r=0.36, p=0.249 b) r=0.52, p=0.038 | WLR R2: a) rr ORR vs. HR OS b) diff ORR vs. diff med OS | b) R2=0.13, p=0.249 b) R2=0.27, p=0.038 | a) Slope -0.113 b) Slope 0.133 | NR | Low | NE |
| Cremolini 2017^31^ | ORR | OS | Colorectal | 2nd | Targeted, not anti-angiogenic | 7* | NR | Pearson (via WLR): a) rr ORR vs. HR OS b) diff ORR vs. diff med OS | a) r=0.44, p=0.274 b) r=0.63, p=0.068 | WLR R2: a) rr ORR vs. HR OS b) diff ORR vs. diff med OS | b) R2=0.20, p=0.274 b) R2=0.40, p=0.068 | a) Slope -0.064 b) Slope 0.143 | NR | Low | NE |
| Johnson 2006^46^ | ORR | OS | Colorectal | 1st | Chemo | 146^†^ | 35337^†^ |  |  | WLSR R2 (diff ORR vs. diff med OS) | R2=0.10, p<0.0001 | Diff med OS = 0.340 + 0.096 * diff ORR | NR | Low | NE |
| Sidhu 2013^73^ | ORR | OS | Colorectal | 1st (most) | Chemo +/- targeted | 24^†^ | 20438^†^ | Correlation (NR): a) OR ORR vs. HR OS b) Diff ORR vs. HR OS c) Ratio ORR vs. HR OS | a) r=0.62 (0.37 to 0.79), p=NR b) r=0.64 (0.39 to 0.79), p=NR c) r=0.52 (0.23 to 0.72), p=NR | LR (unwtd) R2: a) OR ORR vs. HR OS b) Diff ORR vs. HR OS c) Ratio ORR vs. HR OS | a) R2=0.39 (0.13 to 0.62), p=NR b) R2=0.41 (0.15 to 0.63), p=NR c) R2=0.27 (0.05 to 0.52), p=NR |  | NR | Medium | NE |
| Sidhu 2013^73^ | ORR | OS | Colorectal | 1st (most) | Targeted + chemo | 13 | 12060* | Correlation (NR): a) OR ORR vs. HR OS b) Diff ORR vs. HR OS c) Ratio ORR vs. HR OS | a) r=0.50 (0.05 to 0.75), p=NR b) r=0.58 (0.19 to 0.80), p=NR c) r=0.42 (0.00 to 0.71), p=NR | LR (unwtd) R2: a) OR ORR vs. HR OS b) Diff ORR vs. HR OS c) Ratio ORR vs. HR OS | a) R2=0.25 (0.00 to 0.57), p=NR b) R2=0.33 (0.04 to 0.64), p=NR c) R2=0.18 (0.00 to 0.51), p=NR |  | NR | Medium | NE |
| Sidhu 2013^73^ | ORR | OS | Colorectal | 1st (most) | Targeted (anti-EGFR) | 9 | 7792* | Correlation (NR): a) OR ORR vs. HR OS b) Diff ORR vs. HR OS c) Ratio ORR vs. HR OS | a) r=0.67 (0.27 to 0.86), p=NR b) r=0.72 (0.35 to 0.88), p=NR c) r=0.52 (0.00 to 0.79), p=NR | LR (unwtd) R2: a) OR ORR vs. HR OS b) Diff ORR vs. HR OS c) Ratio ORR vs. HR OS | a) R2=0.45 (0.07 to 0.74), p=NR b) R2=0.52 (0.12 to 0.78), p=NR c) R2=0.27 (0.00 to 0.62), p=NR |  | NR | Medium | NE |
| Sidhu 2013^73^ | ORR | OS | Colorectal | 1st (most) | Targeted (anti-EGFR), KRAS non-mutant | 6* | 4916* | Correlation (NR): a) OR ORR vs. HR OS b) Diff ORR vs. HR OS c) Ratio ORR vs. HR OS | a) r=0.68 (0.07 to 0.89), p=NR b) r=0.81 (0.38 to 0.94), p=NR c) r=0.48 (0.00 to 0.82), p=NR | LR (unwtd) R2: a) OR ORR vs. HR OS b) Diff ORR vs. HR OS c) Ratio ORR vs. HR OS | a) R2=0.46 (0.01 to 0.80), p=NR b) R2=0.65 (0.15 to 0.88), p=NR c) R2=0.23 (0.00 to 0.67), p=NR |  | NR | Medium | NE |
| Tang 2007^75^ | ORR | OS | Colorectal | 1st | Chemo | 39 | 18668 | Spearman (diff ORR vs. diff med OS) | rs=0.39 (0.08 to 0.63), p=0.015 |  |  |  | NR | Low | Poor |
| Tsujino 2010^77^ | ORR | OS | Colorectal | NR | Targeted | 7 | NR |  |  | LR (unwtd) R2 (diff ORR vs. HR OS) | R2=0.51, p=0.072 | Slope -0.029 | NR | Medium | NE |
| Blumenthal 2017^22^ | ORR | OS | Lung (NSCLC) | Various | Chemo, immune or targeted | 25 | 20013^†^ |  |  | WLR R2: a) OR ORR vs. HR OS b) 6mo ratio ORR vs. HR OS | a) R2=0.04 (0.0002 to 0.28), p=NR b) R2=0.05 (0.0001 to 0.31), p=NR |  | NR | Low | NE |
| Blumenthal 2015^21^ | ORR | OS | Lung (NSCLC) | Various | Chemo or targeted | 14 | 12567^†^ |  |  | WLR R2 (logOR ORR vs. logHR OS) | R2=0.09 (0 to 0.33), p=NR |  | NR | Low | NE |
| Blumenthal 2015^21^ | ORR | OS | Lung (NSCLC) | Various | Chemo | 11 | 11701^†^ |  |  | WLR R2 (logOR ORR vs. logHR OS) | R2=0.44 (0.08 to 0.80), p=NR |  | NR | Low | NE |
| Hashim 2018^39^ | ORR | OS | Lung (NSCLC) | 2nd + | Various | 140 | 41725 | Correlation (NR) via WLR: a) diff ORR vs. logHR OS b) diff ORR vs. diff med OS | a) r=0.17 (0.00 to 0.38), p=NR b) r=0.18 (0.02 to 0.34), p=0.032 |  |  |  | NA | Low | NE |
| Hashim 2018^39^ | ORR | OS | Lung (NSCLC) | - 2nd + - Phase III | Various | 59 | 32348 | Correlation (NR) via WLR: a) diff ORR vs. logHR OS b) diff ORR vs. diff med OS | a) r=0.37 (0.09 to 0.60), p=NR b) r=0.13 (0.00 to 0.38), p=0.32 |  |  |  | NA | Low | NE |
| Hashim 2018^39^ | ORR | OS | Lung (NSCLC) | - 2nd + - Phase III excl per-protocol crossover | Various | 54 | 30654 | Correlation (NR) via WLR: a) diff ORR vs. logHR OS b) diff ORR vs. diff med OS | a) r=0.40 (0.10 to 0.63), p=NR b) r=0.36 (0.10 to 0.57), p=0.0074 |  |  |  | NA | Low | NE |
| Hashim 2018^39^ | ORR | OS | Lung (NSCLC) | - 2nd + - Phase III excl any crossover | Various | 38 | 22574 | Correlation (NR) via WLR: a) diff ORR vs. logHR OS b) diff ORR vs. diff med OS | a) r=0.52 (0.18 to 0.75), p=NR b) r=0.45 (0.15 to 0.67), p=0.0051 |  |  |  | a) 55% b) NA | Medium | NE |
| Hashim 2018^39^ | ORR | OS | Lung (NSCLC) | - 2nd + - Phase III excl crossover or unbalanced post-progression treatments | Various | 18 | 13349 | Correlation (NR) via WLR: a) diff ORR vs. logHR OS b) diff ORR vs. diff med OS | a) r=0.16 (0.00 to 0.60), p=NR b) r=0.53 (0.08 to 0.80), p=0.024 |  |  |  | a) NA b) 41% | Low | NE |
| Hotta 2015^40^ | ORR | OS | Lung (NSCLC) | Various | Targeted | 18 | 7633^†^ |  |  | WLR R2 (OR ORR vs. HR OS) | R2=0.10, p=NR |  | NR | Low | NE |
| Hotta 2015^40^ | ORR | OS | Lung (NSCLC) | - Various - Molecularly selected | Targeted | 8 | NR |  |  | WLR R2 (OR ORR vs. HR OS) | R2=0.04, p=NR |  | NR | Low | NE |
| Hotta 2015^40^ | ORR | OS | Lung (NSCLC) | - Various - Non-molecularly selected | Targeted | 10 | NR |  |  | WLR R2 (OR ORR vs. HR OS) | R2=0.43, p=NR |  | NR | Low | NE |
| Ito 2019^45^ | ORR | OS | Lung (NSCLC) | Various | Immune checkpoint inhibitors (PD-(L)1) | 6 | 3752^†^ | a) Pearson, wtd b) Spearman, wtd (OR ORR vs. HR OS) | a) r= -0.75, p<0.0001 b) rs= -0.96, p<0.0001 | WLR R2 (OR ORR vs. HR OS) | R2=0.57, p=0.051 |  | NR | Medium | Poor |
| Ito 2019^45^ | ORR | OS | Lung (NSCLC) | - Various - High PD-L1 expression | Immune checkpoint inhibitors (PD-(L)1) | 7 | 1381 | a) Pearson, wtd b) Spearman, wtd (OR ORR vs. HR OS) | a) r= -0.50, p<0.0001 b) rs= -0.21, p<0.0001 | WLR R2 (OR ORR vs. HR OS) | R2=0.25, p=0.253 |  | NR | Low | Fair |
| Johnson 2006^46^ | ORR | OS | Lung (NSCLC) | 1st | Chemo | 191^†^ | 44125^†^ |  |  | WLSR R2 (diff ORR vs. diff med OS) | R2=0.16, p<0.0001 | Diff med OS = -0.048 + 0.090 * diff ORR | NR | Low | NE |
| Nakashima 2016^58^ | ORR | OS | Lung (NSCLC) | 1st | Chemo | 44 | 22709 | Spearman, wtd (lnOR ORR vs. HR OS) | rs=0.57, p=NR | WLSR adj R2 (lnOR ORR vs. lnHR OS) | Adj R2=0.35, p=NR | lnHR OS = -0.023 -0.133 x lnOR ORR | NR | Low | NE |
| Ritchie 2018^65^ | ORR | OS | Lung (NSCLC) | All | Immune checkpoint inhibitors (PD-(L)1 or CTLA4) | 8 | NR | Correlation (NR), wtd (OR ORR vs. HR OS) | r=0.68 (0.08 to 1.10), p=NR |  |  |  | NR | Low | Good |
| Roviello 2017^67^ | ORR | OS | Lung (NSCLC) | Various | Immune checkpoint inhibitors | 7* | 3369* |  |  | WLR R2 (logOR ORR vs. logHR OS) | R2=0.0007 (0.09 to 0.91), p=0.94 |  | NR | Low | NE |
| Tsujino 2010^77^ | ORR | OS | Lung (NSCLC) | NR | Targeted | 5 | NR |  |  | LR (unwtd) R2 (diff ORR vs. HR OS) | R2=0.84, p=0.030 | Slope -0.011 | NR | Medium+ | NE |
| Foster 2011^34^ | ORR | OS | Lung (SCLC) | 1st | Chemo | 3 (32 centres) | 596^†^ | Spearman (logOR ORR vs. logHR OS) | rs=0.52, p=NR | WLSR R2 (logOR ORR vs. logHR OS) | R2=0.21, p=NR |  | NR | Low | NE |
| Hotta 2009^41^ | ORR | OS | Lung (SCLC) | 1st | Chemo | 48 | 8779 |  |  | WLR R2 (rr ORR vs. diff med OS) | R2=0.33, p=NR | Diff med OS = 0.00 + 0.06 * rr ORR | NR | Low | NE |
| Hotta 2009^41^ | ORR | OS | Lung (SCLC) | - 1st - Clear criteria | Chemo | 43 comp | NR |  |  | WLR R2 (rr ORR vs. diff med OS) | R2=0.19, p=NR |  | NR | Low | NE |
| Hotta 2009^41^ | ORR | OS | Lung (SCLC) | - 1st - WHO criteria | Chemo | 23 comp | NR |  |  | WLR R2 (rr ORR vs. diff med OS) | R2=0.13, p=NR |  | NR | Low | NE |
| Hotta 2009^41^ | ORR | OS | Lung (SCLC) | - 1st - Non-WHO criteria | Chemo | 20 comp | NR |  |  | WLR R2 (rr ORR vs. diff med OS) | R2=0.28, p=NR |  | NR | Low | NE |
| Hotta 2009^41^ | ORR | OS | Lung (SCLC) | - 1st - Published 1990-1996 | Chemo | 26 comp | NR |  |  | WLR R2 (rr ORR vs. diff med OS) | R2=0.23, p=NR | Diff med OS = 0.00 + 0.04 * rr ORR | NR | Low | NE |
| Hotta 2009^41^ | ORR | OS | Lung (SCLC) | - 1st - Published 1997-2008 | Chemo | 26 comp | NR |  |  | WLR R2 (rr ORR vs. diff med OS) | R2=0.47, p=NR | Diff med OS = 0.00 + 0.09 * rr ORR | NR | Low | NE |
| Colloca & Venturino 2017^27^ | ORR | OS | Ovarian | 1st | Chemo | 27 | NR | Spearman (diff ORR vs. diff med OS) | rs=0.41, p=0.035 | LR R2 (log RR ORR vs. log HR OS) | R2=0.12, p=0.073 |  | NR | Low | NE |
| Colloca & Venturino 2017^27^ | ORR | OS | Ovarian | - 1st - Published 1990-2002 | Chemo | 13 | NR | Spearman (diff ORR vs. diff med OS) | rs=0.65, p=0.016 | LR R2 (log RR ORR vs. log HR OS) | R2=0.15, p=0.199 |  | NR | Low | NE |
| Colloca & Venturino 2017^27^ | ORR | OS | Ovarian | - 1st - Published 2003-2016 | Chemo | 14 | NR | Spearman (diff ORR vs. diff med OS) | rs= -0.02, p=0.940 | LR R2 (log RR ORR vs. log HR OS) | R2=0.34, p=0.027 |  | NR | Low | NE |
| Siddiqui 2017^72^ | ORR | OS | Ovarian | 2nd + | Chemo | 31^†^ | 9223^†^ |  |  |  |  |  | NR | NE | NE |
| Colloca 2016a^28^ | ORR | OS | Pancreatic | 1st | Gemcitabine + chemo or targeted | 36* | NR | Spearman (diff ORR vs. diff med OS) | rs=0.29, p=0.067 |  |  |  | NR | Low | NE |
| Colloca 2016a^28^ | ORR | OS | Pancreatic | 1st | Gemcitabine + chemo | 22* | NR | Spearman (diff ORR vs. diff med OS) | rs=0.23, p=0.250 | LR R2 (logRR ORR vs. logHR OS) | R2=0.15, p=NR |  | NR | Low | NE |
| Colloca 2016a^28^ | ORR | OS | Pancreatic | 1st | Gemcitabine + targeted | 14* | NR | Spearman (diff ORR vs. diff med OS) | rs=0.55, p=0.035 | LR R2 (logRR ORR vs. logHR OS) | R2=0.28, p=NR |  | NR | Low | NE |
| Hamada 2016^37^ | ORR | OS | Pancreatic | 1st | Chemo | 36 | 15906^†^ | Spearman via WLSR (logOR ORR vs. logHR OS) | rs= -0.16 (-0.27 to -0.05), p=0.007 | WLSR adj R2 (logOR ORR vs. logHR OS) | Adj R2=0.30, p=0.007 |  | NR | Low | Poor |
| Makris 2017^53^ | ORR | OS | Pancreatic (adenocarcinoma) | 1st | Chemo (gemcitabine) | 22* | 10379* | Pearson (log HR OS vs. log OR ORR): a) wtd by sample size b) fixed effect c) random effects | a) r=0.27 (-0.14 to 0.60), p=0.20 b) r=0.52 (0.16 to 0.76), p=0.007 c) r=0.45 (0.07 to 0.72), p=0.02 |  |  |  | NR | Low | NE |
| Makris 2017^53^ | ORR | OS | Pancreatic (adenocarcinoma) | - 1^st^  - No crossover | Chemo (gemcitabine) | 22* | 10379* | Pearson (log HR OS vs. log OR ORR): a) wtd by sample size b) fixed effect c) random effects | a) r= -0.10 (-0.56 to 0.40), p=0.70  b) r=0.16 (-0.34 to 0.60), p=0.53  c) r=0.21 (-0.30 to 0.62), p=0.43 |  |  |  | NR | Low | NE |
| Makris 2017^53^ | ORR | OS | Pancreatic (adenocarcinoma) | - 1^st^  - Crossover <50% | Chemo (gemcitabine) | 22* | 10379* | Pearson (log HR OS vs. log OR ORR): a) wtd by sample size b) fixed effect c) random effects | a) r=0.26 (-0.18 to 0.62), p=0.24  b) r=0.53 (0.15 to 0.78), p=0.009  c) r=0.45 (0.03 to 0.73), p=0.03 |  |  |  | NR | Low | NE |
| Colloca 2016c^30^ | ORR | OS | Prostate | 1st + 2nd | Chemo, hormonal + targeted | 17 | NR | Pearson (diff ORR vs. diff med OS) | r=0.38, p=0.132 | LR R2 (log RR ORR vs. log HR OS) | R2=0.007, p=0.789 |  | NR | Low | NE |
| Colloca 2016c^30^ | ORR | OS | Prostate | - 1st + 2nd - Published 1995-2004 | Chemo, hormonal + targeted | 5 | NR | Pearson (diff ORR vs. diff med OS) | r=0.35, p=0.560 | LR R2 (log RR ORR vs. log HR OS) | R2=0.53, p=0.275 |  | NR | Medium | NE |
| Colloca 2016c^30^ | ORR | OS | Prostate | - 1st + 2nd - Published 2005-2014 | Chemo, hormonal + targeted | 12 | NR | Pearson (diff ORR vs. diff med OS) | r=0.41, p=0.185 | LR R2 (log RR ORR vs. log HR OS) | R2=0.02, p=0.690 |  | NR | Low | NE |
| Delea 2012^32^ | ORR | OS | Renal cell | NR | Cytokine or targeted | 25* | 10943^†^ | Pearson, wtd (ln(rr) ORR vs. -lnHR OS) | r=0.78, p<0.0001 | WLSR adj R2 (ln rr ORR vs. -lnHR OS) | Adj R2=0.59, p<0.0001 | -lnHR OS = -0.11 + 0.30 * lnrr ORR | NR | Medium | NE |
| Petrelli 2013^63^ | ORR | OS | Renal cell | 1st | Targeted | 6^†^ | 3188^†^ | a) Pearson, wtd b) Spearman, wtd (diff med OS vs. diff ORR) | a) r =0.52, p<0.0001 b) rs = 0.49, p<0.0001 | LR | R2=0.27, p=NR |  | NR | Low | Fair |
| Tanaka 2019^74^ | ORR | OS | Soft tissue sarcoma | 1st | Chemo | 27^†^ | 6156^†^ | Kendall's Tau (logOR ORR vs. logHR OS) | τ=0.41, p=NR | Regression (NR) R2 (logOR ORR vs. logHR OS) | R2=0.28 (0.02 to 0.54), p=NR |  | NR | Low | NE |
| Zer 2016^80^ | ORR | OS | Soft tissue sarcoma | All | Systemic | 52^†^ | 9762^†^ | Correlation (NR) via WLR (OR ORR vs. HR OS) | r=0.51, p=NR |  |  |  | NR | Low | NE |
| Kaufman 2018^47^ | ORR | OS | Various solid tumours | Various | Immune checkpoint inhibitors + chemo | 27^†^ | 10300^†^ |  |  | WLR adj R2 (OR ORR vs. HR OS) | Adj R2= -0.07, p=0.866 |  | NR | NE | NE |
| Kaufman 2018^47^ | ORR | OS | Various solid tumours | Various | Immune checkpoint inhibitors alone | NR | NR |  |  | WLR adj R2 (OR ORR vs. HR OS) | Adj R2= -0.08, p=0.799 |  | NR | NE | NE |
| Mushti 2018^57^ | ORR | OS | Various solid tumours | NR | Immune checkpoint inhibitors (PD-(L)1) | 13 | 6722 |  |  | WLR R2 (OR ORR vs. HR OS) | R2=0.13, p=NR |  | NR | Low | NE |
| Nie 2019^60^ | ORR | OS | Various solid tumours | Various | Immune checkpoint inhibitors (PD-(L)1) | 43^†^ | 15088^†^ |  |  | WLR R2 (lnOR ORR vs. lnHR OS) | R2=0.10, p=0.053 |  | NR | Low | Poor |
| Ritchie 2018^65^ | ORR | OS | Various solid tumours | All | Immune checkpoint inhibitors (PD-(L)1 or CTLA4) | 20^†^ | 10828^†^ | Correlation (NR), wtd (OR ORR vs. HR OS) | r=0.57 (0.23 to 0.89), p=NR |  |  |  | NR | Low | Poor |
| Roviello 2017^67^ | ORR | OS | Various solid tumours | Various | Immune checkpoint inhibitors | 17^†^ | 8994^†^ |  |  | WLR R2 (logOR ORR vs. logHR OS) | R2=0.47 (0.03 to 0.77), p=0.001 | logHR OS = -0.1329 -0.2575 * logOR ORR | NR | Low | NE |
| Roviello 2017^67^ | ORR | OS | Various solid tumours | Various | Immune checkpoint inhibitors (CTLA-4) | 17^†^ | 8994^†^ |  |  | WLR R2 (logOR ORR vs. logHR OS) | R2=0.00 (0.00 to 0.97), p=0.96 |  | NR | Low | NE |
| Roviello 2017^67^ | ORR | OS | Various solid tumours | Various | Immune checkpoint inhibitors (PD-(L)1) | 17^†^ | 8994^†^ |  |  | WLR R2 (logOR ORR vs. logHR OS) | R2=0.18 (0.00 to 0.97), p=0.17 |  | NR | Low | NE |
| Tsujino 2010^77^ | ORR | OS | Various solid tumours | NR | Targeted | 18 | NR |  |  | LR (unwtd) R2 (diff ORR vs. HR OS) | R2=0.47, p=0.002 | Slope -0.016 | 21% | Low | NE |
| Wilkerson+Fojo 2009^79^ | ORR | OS | Various solid tumours | NR | NR | 66^†^ | NR |  |  | LR (unwtd R2): a) diff ORR vs. HR OS b) diff ORR vs. diff med OS | a) R2=0.37, p<0.0001 b) R2=0.34, p<0.0001 |  | NR | Low | NE |
| **CR vs. PFS** | | | | | | | | | | | | | | | |
| Lee 2011^48^ | CR | PFS | NHL (aggressive) | 1st | Chemo | 12^†^ | NR | Spearman (diff CR vs. diff 3yr PFS) | rs=0.63 (0.21 to 0.84), p=0.005 |  |  |  | NR | Medium | NE |
| Lee 2011^48^ | CR | PFS | NHL (indolent) | 1st | Chemo | 6^†^ | NR | Spearman (diff CR vs. diff 3yr PFS) | rs=0.41 (-0.52 to 0.88), p=0.35 |  |  |  | NR | Medium | NE |
| Shi 2017^69^ | CR | PFS | NHL (indolent; follicular) | 1st | Chemo or immuno (induction or maintenance) | 13 | 3837 |  |  | a) WLSR R2 b) Bivariate Plackett copula model (logOR CR 30mo vs. logHR PFS) | a) R2WLS=0.88 (0.77 to 0.96), p=NR b) R2Copula=0.86 (0.72 to 1.00), p=NR | logHR PFS = -0.093 - 0.636 * logOR CR 30mo | 1.56 | Medium+ | NE |
| Shi 2017^69^ | CR | PFS | NHL (indolent; follicular) | 1st | Rituximab-based (induction or maintenance) | 9 | 2851 |  |  | a) WLSR R2 b) Bivariate Plackett copula model (logOR CR 30mo vs. logHR PFS) | a) R2WLS=0.85 (0.62 to 0.97), p=NR b) R2Copula=0.80 (0.56 to 1.00), p=NR |  | NR | Medium+ | NE |
| Shi 2017^69^ | CR | PFS | NHL (indolent; follicular) | 1st | Non-rituximab-based (induction or maintenance) | 4 | 986 |  |  | a) WLSR R2 b) Bivariate Plackett copula model (logOR CR 30mo vs. logHR PFS) | a) R2WLS=0.91 (0.05 to 1.00), p=NR b) R2Copula=0.96 (0.90 to 1.00), p=NR |  | NR | Medium+ | NE |
| Shi 2017^69^ | CR | PFS | NHL (indolent; follicular) | 1st | Induction | 8 | 2207 |  |  | a) WLSR R2 b) Bivariate Plackett copula model (logOR CR 30mo vs. logHR PFS) | a) R2WLS=0.89 (0.75 to 0.98), p=NR b) R2Copula=0.89 (0.74 to 1.00), p=NR |  | NR | Medium+ | NE |
| Shi 2017^69^ | CR | PFS | NHL (indolent; follicular) | 1st | Maintenance | 5 | 1630 |  |  | wtd least squares (reported as R2WLS) and bivariate Plackett copula model (reported as R2copula), CR30 vs PFS | a) R2WLS=0.93 (0.84 to 1.00), p=NR b) R2Copula=0.89 (0.71 to 1.00), p=NR |  | NR | Medium+ | NE |
| Shi 2017^69^ | CR | PFS | NHL (indolent; follicular) | - 1st - High FLIPI score | Chemo or immuno (induction or maintenance) | 9 | 1415 |  |  | a) WLSR R2 b) Bivariate Plackett copula model (logOR CR 30mo vs. logHR PFS) | a) R2WLS=0.87 (0.68 to 0.98), p=NR b) R2Copula=0.73 (0.42 to 1.00), p=NR |  | NR | Medium+ | NE |
| Shi 2017^69^ | CR | PFS | NHL (indolent; follicular) | - 1st - Low to intermediate FLIPI score | Chemo or immuno (induction or maintenance) | 10 | 1882 |  |  | a) WLSR R2 b) Bivariate Plackett copula model (logOR CR 30mo vs. logHR PFS) | a) R2WLS=0.45 (0.02 to 0.93), p=NR b) R2Copula=0.57 (0.17 to 0.97), p=NR |  | NR | Low | NE |
| Shi 2017^69^ | CR | PFS | NHL (indolent; follicular) | 1st | Chemo or immuno (induction or maintenance) | 11 | 2728 |  |  | a) WLSR R2 b) Bivariate Plackett copula model (logOR CR 24mo vs. logHR PFS) | a) R2WLS=0.84 (0.63 to 0.95), p=NR b) R2Copula=0.67 (0.35 to 0.99), p=NR | logHR PFS = 0.043 - 0.726 * logOR CR24mo | NR | Medium+ | NE |
| Shi 2017^69^ | CR | PFS | NHL (indolent; follicular) | - 1st - Stage IV | Chemo or immuno (induction or maintenance) | NR | 2585 |  |  | a) WLSR R2 b) Bivariate Plackett copula model (logOR CR 30mo vs. logHR PFS) | a) R2WLS=0.92 (0.85 to 0.97), p=NR b) R2Copula=0.94 (0.87 to 1.00), p=NR |  | NR | Medium+ | NE |
| Colloca & Venturino 2017^27^ | CR | PFS | Ovarian | 1st | Chemo | 12 | NR | Spearman (diff RR vs. diff med PFS) | rs=0.19, p=0.555 |  |  |  | NR | Low | NE |
| **CR vs. OS** | | | | | | | | | | | | | | | |
| Hackshaw 2005^36^ | CR | OS | Breast | 1st | Chemo | 41* | 9163^†^ |  |  | WLR R2 (logOR CR vs. logHR OS) | R2=0.12, p=0.02 | logHR OS = -0.0097 + 0.13 * logOR CR  Slope 0.13 | NR | Low | NE |
| Hackshaw 2005^36^ | CR | OS | Breast | - 1st - Recruited pre-1990 | Chemo | 26* | 5244^†^ |  |  | WLR R2 (logOR CR vs. logHR OS) | R2=0.05, p=0.24 | Slope 0.09 | NR | Low | NE |
| Hackshaw 2005^36^ | CR | OS | Breast | - 1st - Recruited 1990 or after | Chemo | 15* | 3919^†^ |  |  | WLR R2 (logOR CR vs. logHR OS) | R2=0.36, p=0.01 | Slope 0.16 | NR | Low | NE |
| Foster 2011^34^ | CR | OS | Lung (SCLC) | 1st | Chemo | 3 (32 centres) | 596^†^ | Spearman (logOR CR vs. logHR OS) | rs=0.50, p=NR | WLSR R2 (logOR CR vs. logHR OS) | R2=0.48, p=NR |  | NR | Low | NE |
| Lee 2011^48^ | CR | OS | NHL (aggressive) | 1st | Chemo | 36^†^ | 16103^†^ | Spearman: a) diff CR vs. diff 3yr OS b) diff CR vs. diff 5yr OS | a) rs=0.58 (0.29 to 0.77), p=0.004 b) rs=0.50 (0.23 to 0.74), p=0.01 |  |  |  | NR | Medium | NE |
| Lee 2011^48^ | CR | OS | NHL (indolent) | 1st | Chemo | 15^†^ | 5128^†^ | Spearman: a) diff CR vs. diff 3yr OS b) diff CR vs. diff 5yr OS | a) rs=0.41 (-0.10 to 0.74), p=0.098 b) rs=0.21 (-0.34 to 0.50), p=0.44 |  |  |  | NR | Medium | NE |
| Colloca & Venturino 2017^27^ | CR | OS | Ovarian | 1st | Chemo | 12 | NR | Spearman (diff pCR vs. diff med OS) | rs=0.42, p=0.180 |  |  |  | NR | Low | NE |
| **DoR vs. OS** | | | | | | | | | | | | | | | |
| Colloca 2016b^29^ | DoR | OS | Colorectal | 1st | Bevacizumab + chemo | 5 | NR | Spearman (diff med DoR vs. diff med OS) | rs=0.70, p=0.188 |  |  |  | NR | Medium | NE |
| Colloca 2016a^28^ | DoR | OS | Pancreatic | 1st | Gemcitabine + chemo or targeted | 7^†^ | NR | Spearman (diff med DoR vs. diff med OS) | rs=0.76, p=0.049 |  |  |  | NR | Medium | NE |
| Colloca 2016a^28^ | DoR | OS | Pancreatic | 1st | Gemcitabine + chemo | 3^†^ | NR | Spearman (diff med DoR vs. diff med OS) | rs=0.50, p=0.667 |  |  |  | NR | Low | NE |
| Colloca 2016a^28^ | DoR | OS | Pancreatic | 1st | Gemcitabine + targeted | 4^†^ | NR | Spearman (diff med DoR vs. diff med OS) | rs=0.40, p=0.600 |  |  |  | NR | Low | NE |
| *Calculated from reported data. ^†^Unclear for individual subgroups.  adj, adjusted; BSES2, Biomarker-Surrogate Evaluation Schema criteria 2; chemo, chemotherapy; CI, confidence interval; CR, complete response; diff, difference;; DoR, duration of response; FO, final outcome; HR, hazard ratio; IQWiG, Institute of Quality and Efficiency in Health Care; ln, natural logarithm; log, logarithm; LR, linear regression; med, median; mo, months; NE, not estimable; NHL, non-Hodgkin’s lymphoma; NR, not reported; NSCLC, non-small cell lung cancer; OR, odds ratio; ORR, overall response rate (ORR=PR+CR); OS, overall survival; PFS, progression-free survival; r, Pearson correlation; R2, regression coefficient of determination; rs, Spearman rank correlation; rr, relative risk; SCLC, small cell lung cancer; SO, surrogate outcome; STE, surrogate threshold effect; unwtd, unweighted; wtd, weighted; WLR, weighted linear regression; WLSR, weighted least squares regression. | | | | | | | | | | | | | | | |

Supplementary Table 9: Influence of clinical and study factors on association between ORR and OS

| **Disease and factor comparison** | **Absolute association (r)** | | **Treatment effect association (R^2^)** | |
| --- | --- | --- | --- | --- |
|  | **Range Factor A** | **Range Factor B** | **Range Factor A** | **Range Factor B** |
| **AML** | | | | |
| Treatment line: (A) 1st line vs (B) subsequent line | *INSUFFICIENT DATA* | *INSUFFICIENT DATA* | *INSUFFICIENT DATA* | *INSUFFICIENT DATA* |
| Treatment type: (A) targeted vs (B) systemic | *INSUFFICIENT DATA* | *INSUFFICIENT DATA* | *INSUFFICIENT DATA* | *INSUFFICIENT DATA* |
| Response criteria: (A) RECIST vs (B) WHO | *INSUFFICIENT DATA* | *INSUFFICIENT DATA* | *INSUFFICIENT DATA* | *INSUFFICIENT DATA* |
| OS adjustment: (A) adjusted vs (B) unadjusted | *INSUFFICIENT DATA* | *INSUFFICIENT DATA* | *INSUFFICIENT DATA* | *INSUFFICIENT DATA* |
| Data type: (A) Aggregate vs (B) IPD | *INSUFFICIENT DATA* | *INSUFFICIENT DATA* | *INSUFFICIENT DATA* | *INSUFFICIENT DATA* |
| **Biliary tract** | | | | |
| Treatment line: (A) 1st line vs (B) subsequent line | *INSUFFICIENT DATA* | *INSUFFICIENT DATA* | *INSUFFICIENT DATA* | *INSUFFICIENT DATA* |
| Treatment type: (A) targeted vs (B) systemic | *INSUFFICIENT DATA* | *INSUFFICIENT DATA* | **0.43 ^56^** | **0.29 to 0.39 ^56^** |
| Response criteria: (A) RECIST vs (B) WHO | *INSUFFICIENT DATA* | *INSUFFICIENT DATA* | *INSUFFICIENT DATA* | *INSUFFICIENT DATA* |
| OS adjustment: (A) adjusted vs (B) unadjusted | *INSUFFICIENT DATA* | *INSUFFICIENT DATA* | *INSUFFICIENT DATA* | *INSUFFICIENT DATA* |
| Data type: (A) Aggregate vs (B) IPD | *INSUFFICIENT DATA* | *INSUFFICIENT DATA* | *INSUFFICIENT DATA* | *INSUFFICIENT DATA* |
| **Breast** | | | | |
| Treatment line: (A) 1st line vs (B) subsequent line | **0.61 ^64^** | **-0.10 to 1.00 ^51^** | *INSUFFICIENT DATA* | *INSUFFICIENT DATA* |
| Treatment type: (A) targeted vs (B) systemic | **0.61 ^64^** | **-0.10 to 1.00 ^51^** | *INSUFFICIENT DATA* | *INSUFFICIENT DATA* |
| Response criteria: (A) RECIST vs (B) WHO | *INSUFFICIENT DATA* | *INSUFFICIENT DATA* | *INSUFFICIENT DATA* | *INSUFFICIENT DATA* |
| OS adjustment: (A) adjusted vs (B) unadjusted | *INSUFFICIENT DATA* | *INSUFFICIENT DATA* | *INSUFFICIENT DATA* | *INSUFFICIENT DATA* |
| Data type: (A) Aggregate vs (B) IPD | *INSUFFICIENT DATA* | *INSUFFICIENT DATA* | **0.26 to 0.41 ^36^** | **0.10 to 0.20^23^** |
| **Colorectal** | | | | |
| Treatment line: (A) 1st line vs (B) subsequent line | **0.41 to 0.59 ^52,75^** | **0.58 ^35^** | **0.10 to 0.58 ^25,29,46^** | **0.03 to 0.40 ^31^** |
| Treatment type: (A) targeted vs (B) systemic | *INSUFFICIENT DATA* | *INSUFFICIENT DATA* | **0.03 to 0.65 ^29,31,73,77^** | **0.06 to 0.40 ^25,26,46^** |
| Response criteria: (A) RECIST vs (B) WHO | *INSUFFICIENT DATA* | *INSUFFICIENT DATA* | **0.58 ^29^** | **0.38 ^25^** |
| OS adjustment: (A) adjusted vs (B) unadjusted | *INSUFFICIENT DATA* | *INSUFFICIENT DATA* | **0.4 ^26^** | **0.03 to 0.65 ^25,26,29,31,46,73,77^** |
| Data type: (A) Aggregate vs (B) IPD | *INSUFFICIENT DATA* | *INSUFFICIENT DATA* | **0.03 to 0.65 ^26,29,31,46,73,77^** | **0.38 ^25^** |
| **Gastric and gastroesophageal** | | | | |
| Treatment line: (A) 1st line vs (B) subsequent line | **0.18 to 0.47 ^42^** | **0.38 ^70^** | *INSUFFICIENT DATA* | *INSUFFICIENT DATA* |
| Treatment type: (A) targeted vs (B) systemic | **0.86 ^61^** | **0.18 to 0.47 ^42,70^** | *INSUFFICIENT DATA* | *INSUFFICIENT DATA* |
| Response criteria: (A) RECIST vs (B) WHO | *INSUFFICIENT DATA* | *INSUFFICIENT DATA* | *INSUFFICIENT DATA* | *INSUFFICIENT DATA* |
| OS adjustment: (A) adjusted vs (B) unadjusted | *INSUFFICIENT DATA* | *INSUFFICIENT DATA* | *INSUFFICIENT DATA* | *INSUFFICIENT DATA* |
| Data type: (A) Aggregate vs (B) IPD | *INSUFFICIENT DATA* | *INSUFFICIENT DATA* | *INSUFFICIENT DATA* | *INSUFFICIENT DATA* |
| **Glioblastoma** | | | | |
| Treatment line: (A) 1st line vs (B) subsequent line | *INSUFFICIENT DATA* | *INSUFFICIENT DATA* | *INSUFFICIENT DATA* | *INSUFFICIENT DATA* |
| Treatment type: (A) targeted vs (B) systemic | *INSUFFICIENT DATA* | *INSUFFICIENT DATA* | *INSUFFICIENT DATA* | *INSUFFICIENT DATA* |
| Response criteria: (A) RECIST vs (B) WHO | *INSUFFICIENT DATA* | *INSUFFICIENT DATA* | *INSUFFICIENT DATA* | *INSUFFICIENT DATA* |
| OS adjustment: (A) adjusted vs (B) unadjusted | *INSUFFICIENT DATA* | *INSUFFICIENT DATA* | *INSUFFICIENT DATA* | *INSUFFICIENT DATA* |
| Data type: (A) Aggregate vs (B) IPD | *INSUFFICIENT DATA* | *INSUFFICIENT DATA* | *INSUFFICIENT DATA* | *INSUFFICIENT DATA* |
| **Neuroendocrine** | | | | |
| Treatment line: (A) 1st line vs (B) subsequent line | *INSUFFICIENT DATA* | *INSUFFICIENT DATA* | *INSUFFICIENT DATA* | *INSUFFICIENT DATA* |
| Treatment type: (A) targeted vs (B) systemic | *INSUFFICIENT DATA* | *INSUFFICIENT DATA* | *INSUFFICIENT DATA* | *INSUFFICIENT DATA* |
| Response criteria: (A) RECIST vs (B) WHO | *INSUFFICIENT DATA* | *INSUFFICIENT DATA* | *INSUFFICIENT DATA* | *INSUFFICIENT DATA* |
| OS adjustment: (A) adjusted vs (B) unadjusted | *INSUFFICIENT DATA* | *INSUFFICIENT DATA* | *INSUFFICIENT DATA* | *INSUFFICIENT DATA* |
| Data type: (A) Aggregate vs (B) IPD | *INSUFFICIENT DATA* | *INSUFFICIENT DATA* | *INSUFFICIENT DATA* | *INSUFFICIENT DATA* |
| **NSCLC** | | | | |
| Treatment line: (A) 1st line vs (B) subsequent line | *INSUFFICIENT DATA* | *INSUFFICIENT DATA* | **0.16 to 0.35 ^46,58^** | **0.03 to 0.27 ^39^** |
| Treatment type: (A) targeted vs (B) systemic | **-0.02 to 0.92 ^45,49,50,65,71^** | **0.41 to 0.62 ^68,71^** | **0.0007 to 0.84 ^40,45,65,67,77^** | **0.16 to 0.44 ^21,46,58^** |
| Response criteria: (A) RECIST vs (B) WHO | **0.52 ^49^** | **0.62 ^68^** | *INSUFFICIENT DATA* | *INSUFFICIENT DATA* |
| OS adjustment: (A) adjusted vs (B) unadjusted | *INSUFFICIENT DATA* | *INSUFFICIENT DATA* | **0.03 to 0.27 ^39^** | **0.0007 to 0.84 ^21,22,39,40,45,46,58,65,67,77^** |
| Data type: (A) Aggregate vs (B) IPD | *INSUFFICIENT DATA* | *INSUFFICIENT DATA* | *INSUFFICIENT DATA* | *INSUFFICIENT DATA* |
| **Ovarian** | | | | |
| Treatment line: (A) 1st line vs (B) subsequent line | *INSUFFICIENT DATA* | *INSUFFICIENT DATA* | *INSUFFICIENT DATA* | *INSUFFICIENT DATA* |
| Treatment type: (A) targeted vs (B) systemic | *INSUFFICIENT DATA* | *INSUFFICIENT DATA* | *INSUFFICIENT DATA* | *INSUFFICIENT DATA* |
| Response criteria: (A) RECIST vs (B) WHO | *INSUFFICIENT DATA* | *INSUFFICIENT DATA* | *INSUFFICIENT DATA* | *INSUFFICIENT DATA* |
| OS adjustment: (A) adjusted vs (B) unadjusted | *INSUFFICIENT DATA* | *INSUFFICIENT DATA* | *INSUFFICIENT DATA* | *INSUFFICIENT DATA* |
| Data type: (A) Aggregate vs (B) IPD | **0.82 ^72^** | **0.56 ^66^** | *INSUFFICIENT DATA* | *INSUFFICIENT DATA* |
| **Pancreatic / adenocarcinoma** | | | | |
| Treatment line: (A) 1st line vs (B) subsequent line | *INSUFFICIENT DATA* | *INSUFFICIENT DATA* | *INSUFFICIENT DATA* | *INSUFFICIENT DATA* |
| Treatment type: (A) targeted vs (B) systemic | *INSUFFICIENT DATA* | *INSUFFICIENT DATA* | **0.28 ^28^** | **0.01 to 0.30 ^28,37,53^** |
| Response criteria: (A) RECIST vs (B) WHO | *INSUFFICIENT DATA* | *INSUFFICIENT DATA* | *INSUFFICIENT DATA* | *INSUFFICIENT DATA* |
| OS adjustment: (A) adjusted vs (B) unadjusted | *INSUFFICIENT DATA* | *INSUFFICIENT DATA* | **0.01 to 0.04 ^53^** | **0.07 to 0.30 ^28,37,53^** |
| Data type: (A) Aggregate vs (B) IPD | *INSUFFICIENT DATA* | *INSUFFICIENT DATA* | *INSUFFICIENT DATA* | *INSUFFICIENT DATA* |
| **Prostate** | | | | |
| Treatment line: (A) 1st line vs (B) subsequent line | *INSUFFICIENT DATA* | *INSUFFICIENT DATA* | *INSUFFICIENT DATA* | *INSUFFICIENT DATA* |
| Treatment type: (A) targeted vs (B) systemic | *INSUFFICIENT DATA* | *INSUFFICIENT DATA* | *INSUFFICIENT DATA* | *INSUFFICIENT DATA* |
| Response criteria: (A) RECIST vs (B) WHO | *INSUFFICIENT DATA* | *INSUFFICIENT DATA* | *INSUFFICIENT DATA* | *INSUFFICIENT DATA* |
| OS adjustment: (A) adjusted vs (B) unadjusted | *INSUFFICIENT DATA* | *INSUFFICIENT DATA* | *INSUFFICIENT DATA* | *INSUFFICIENT DATA* |
| Data type: (A) Aggregate vs (B) IPD | *INSUFFICIENT DATA* | *INSUFFICIENT DATA* | *INSUFFICIENT DATA* | *INSUFFICIENT DATA* |
| **Renal / renal cell** | | | | |
| Treatment line: (A) 1st line vs (B) subsequent line | *INSUFFICIENT DATA* | *INSUFFICIENT DATA* | *INSUFFICIENT DATA* | *INSUFFICIENT DATA* |
| Treatment type: (A) targeted vs (B) systemic | *INSUFFICIENT DATA* | *INSUFFICIENT DATA* | *INSUFFICIENT DATA* | *INSUFFICIENT DATA* |
| Response criteria: (A) RECIST vs (B) WHO | *INSUFFICIENT DATA* | *INSUFFICIENT DATA* | *INSUFFICIENT DATA* | *INSUFFICIENT DATA* |
| OS adjustment: (A) adjusted vs (B) unadjusted | *INSUFFICIENT DATA* | *INSUFFICIENT DATA* | *INSUFFICIENT DATA* | *INSUFFICIENT DATA* |
| Data type: (A) Aggregate vs (B) IPD | *INSUFFICIENT DATA* | *INSUFFICIENT DATA* | *INSUFFICIENT DATA* | *INSUFFICIENT DATA* |
| **SCLC** | | | | |
| Treatment line: (A) 1st line vs (B) subsequent line | *INSUFFICIENT DATA* | *INSUFFICIENT DATA* | *INSUFFICIENT DATA* | *INSUFFICIENT DATA* |
| Treatment type: (A) targeted vs (B) systemic | *INSUFFICIENT DATA* | *INSUFFICIENT DATA* | *INSUFFICIENT DATA* | *INSUFFICIENT DATA* |
| Response criteria: (A) RECIST vs (B) WHO | *INSUFFICIENT DATA* | *INSUFFICIENT DATA* | *INSUFFICIENT DATA* | *INSUFFICIENT DATA* |
| OS adjustment: (A) adjusted vs (B) unadjusted | *INSUFFICIENT DATA* | *INSUFFICIENT DATA* | *INSUFFICIENT DATA* | *INSUFFICIENT DATA* |
| Data type: (A) Aggregate vs (B) IPD | *INSUFFICIENT DATA* | *INSUFFICIENT DATA* | *INSUFFICIENT DATA* | *INSUFFICIENT DATA* |
| **Soft tissue sarcoma** | | | | |
| Treatment line: (A) 1st line vs (B) subsequent line | *INSUFFICIENT DATA* | *INSUFFICIENT DATA* | *INSUFFICIENT DATA* | *INSUFFICIENT DATA* |
| Treatment type: (A) targeted vs (B) systemic | *INSUFFICIENT DATA* | *INSUFFICIENT DATA* | *INSUFFICIENT DATA* | *INSUFFICIENT DATA* |
| Response criteria: (A) RECIST vs (B) WHO | *INSUFFICIENT DATA* | *INSUFFICIENT DATA* | *INSUFFICIENT DATA* | *INSUFFICIENT DATA* |
| OS adjustment: (A) adjusted vs (B) unadjusted | *INSUFFICIENT DATA* | *INSUFFICIENT DATA* | *INSUFFICIENT DATA* | *INSUFFICIENT DATA* |
| Data type: (A) Aggregate vs (B) IPD | *INSUFFICIENT DATA* | *INSUFFICIENT DATA* | *INSUFFICIENT DATA* | *INSUFFICIENT DATA* |
| **Unknown primary** | | | | |
| Treatment line: (A) 1st line vs (B) subsequent line | *INSUFFICIENT DATA* | *INSUFFICIENT DATA* | *INSUFFICIENT DATA* | *INSUFFICIENT DATA* |
| Treatment type: (A) targeted vs (B) systemic | *INSUFFICIENT DATA* | *INSUFFICIENT DATA* | *INSUFFICIENT DATA* | *INSUFFICIENT DATA* |
| Response criteria: (A) RECIST vs (B) WHO | *INSUFFICIENT DATA* | *INSUFFICIENT DATA* | *INSUFFICIENT DATA* | *INSUFFICIENT DATA* |
| OS adjustment: (A) adjusted vs (B) unadjusted | *INSUFFICIENT DATA* | *INSUFFICIENT DATA* | *INSUFFICIENT DATA* | *INSUFFICIENT DATA* |
| Data type: (A) Aggregate vs (B) IPD | *INSUFFICIENT DATA* | *INSUFFICIENT DATA* | *INSUFFICIENT DATA* | *INSUFFICIENT DATA* |
| **Urothelial** | | | | |
| Treatment line: (A) 1st line vs (B) subsequent line | *INSUFFICIENT DATA* | *INSUFFICIENT DATA* | *INSUFFICIENT DATA* | *INSUFFICIENT DATA* |
| Treatment type: (A) targeted vs (B) systemic | ***-0.12 ^18^*** | ***-0.02 to 0.78 ^19^*** | *INSUFFICIENT DATA* | *INSUFFICIENT DATA* |
| Response criteria: (A) RECIST vs (B) WHO | *INSUFFICIENT DATA* | *INSUFFICIENT DATA* | *INSUFFICIENT DATA* | *INSUFFICIENT DATA* |
| OS adjustment: (A) adjusted vs (B) unadjusted | *INSUFFICIENT DATA* | *INSUFFICIENT DATA* | *INSUFFICIENT DATA* | *INSUFFICIENT DATA* |
| Data type: (A) Aggregate vs (B) IPD | *INSUFFICIENT DATA* | *INSUFFICIENT DATA* | *INSUFFICIENT DATA* | *INSUFFICIENT DATA* |
| AML, acute myeloid leukaemia; IPD, individual patient data; NSCLC, non-small cell lung cancer; r, correlation coefficient (e.g. Pearson or Spearman); R^2^, regression coefficient of determination; SCLC, small cell lung cancer. | | | | |

Supplementary Table 10: Regression equations for absolute (individual-level) associations

| **Surrogate relationship** | **Cancer types and references** | **Surrogate** | **Final** | **Intercept** | **Slope** |  |
| --- | --- | --- | --- | --- | --- | --- |
| **ORR to PFS** | Colorectal^52^ | ORR | Median PFS | 3.20 | 0.10 |  |
|  | Lung (NSCLC)^76^ | ORR | Median PFS | NR | 0.07 |  |
|  | Ovarian^72^ | ORR | Median PFS | 2.59 | 0.12 |  |
|  | NHL^54^ | log odds ORR | log median PFS | 1.97 | 0.41 |  |
| **ORR to TTP** | Gastric^42^ | ORR | Median TTP | 1.73 | 0.09 |  |
| **ORR to OS** | Colorectal^52^ | ORR | Median OS | 10.45 | 0.09 |  |
|  | Lung (NSCLC)^76^ | ORR | Median OS | NR | 0.26 |  |
|  | Ovarian^72^ | ORR | Median OS | 9.48 | 0.28 |  |
|  | Gastric^42^ | ORR | Median OS | 5.89 | 0.08 |  |
| **CR to PFS** | NHL^81^ | CR | Median PFS | 0.83 | 0.46 |  |
|  | NHL^54^ | log odds CR | log median PFS | 2.38 | 0.34 |  |
| CR, complete response; NHL, non-Hodgkin’s lymphoma; NSCLC, non-small cell lung cancer; ORR, overall response rate; OS, overall survival; PFS, progression-free survival; TTP, time to progression; VGPR, very good partial response. | | | | | | |

Supplementary Table 11: Regression equations for treatment effect (trial-level) associations

| **Surrogate relationship** | **Cancer types and refs** | **Subgroup** | **Based on difference in response** | | | | | **Based on relative risk or odds ratio for response** | | | | |  |
| --- | --- | --- | --- | --- | --- | --- | --- | --- | --- | --- | --- | --- | --- |
|  |  |  | **Surrogate** | **Final** | **Intercept** | **Slope** | **Surrogate** | | **Final** | **Intercept** | **Slope** |  |  |
| **ORR to PFS** | Lung (NSCLC)^77^ |  | Diff ORR | HR PFS | NR | -0.02 |  | |  |  |  |  |  |
|  | Colorectal^77^ |  | Diff ORR | HR PFS | NR | -0.04 |  | |  |  |  |  |  |
|  | Various^77^ |  | Diff ORR | HR PFS | NR | -0.02 |  | |  |  |  |  |  |
|  | Colorectal^26,33^ |  |  |  |  |  | logOR ORR | | logHR PFS | -0.05 | -0.32 |  |  |
|  | Breast^24^ |  |  |  |  |  | logOR ORR | | logHR PFS | 0.10 | 0.50 |  |  |
|  | Various (immuno)^67^ |  |  |  |  |  | logOR ORR | | logHR PFS | -0.13 | -0.24 |  |  |
| **ORR to OS** | Colorectal^31^ | - All  - Anti-angio  - Non-anti-angio | Diff ORR | Diff median OS | NR | 0.07 0.13 0.14 |  | |  |  |  |  |  |
|  | Colorectal^46^ |  | Diff ORR | Diff median OS | 0.34 | 0.10 |  | |  |  |  |  |  |
|  | Lung (NSCLC)^46^ |  | Diff ORR | Diff median OS | -0.05 | 0.09 |  | |  |  |  |  |  |
|  | Colorectal^77^ |  | Diff ORR | HR OS | NR | -0.03 |  | |  |  |  |  |  |
|  | Lung (NSCLC)^77^ |  | Diff ORR | HR OS | NR | -0.01 |  | |  |  |  |  |  |
|  | Various^77^ |  | Diff ORR | HR OS | NR | -0.02 |  | |  |  |  |  |  |
|  | Colorectal^26,33^ | - All  - No crossover |  |  |  |  | logOR ORR | | logHR OS | -0.03  -0.04 | -0.05  -0.10 |  |  |
|  | Breast^36^ | - All  - Recr. pre-1990  - Recr. 1990 or after |  |  |  |  | logOR ORR | | logHR OS | -0.01  NR  NR | 0.28  0.28  0.24 |  |  |
|  | Lung (NSCLC)^58^ |  |  |  |  |  | lnOR ORR | | lnHR OS | -0.02 | -0.13 |  |  |
|  | Various (immuno)^67^ |  |  |  |  |  | logOR ORR | | logHR OS | -0.13 | -0.26 |  |  |
|  | Colorectal^31^ | - All  - Anti-angio  - Non-anti-angio |  |  |  |  | rr ORR | | HR OS | NR | -0.03  -0.11  -0.06 |  |  |
|  | Renal cell^32^ |  |  |  |  |  | ln rr ORR | | -lnHR OS | -0.11 | 0.30 |  |  |
|  | Biliary tract^56^ | - Chemo  - Gemcitabine  - Targeted |  |  |  |  | Ratio of ORR | | log ratio of median OS | 0.01  0.02  0.12 | 0.28  0.27  0.16 |  |  |
|  | Lung (SCLC)^41^ | - All  - Pub. 1990-1996  - Pub. 1997-2008 |  |  |  |  | rr ORR | | Diff median OS | 0.00  0.00  0.00 | 0.06  0.04  0.09 |  |  |
| **CR to PFS** | NHL^69^ |  |  |  |  |  | logOR CR 30mo | | logHR PFS | -0.09 | -0.64 |  |  |
|  | NHL^69^ |  |  |  |  |  | logOR CR 24mo | | logHR PFS | 0.04 | -0.73 |  |  |
| **CR to OS** | Breast^36^ | - All  - Recr. pre-1990  - Recr. 1990 or after |  |  |  |  | logOR CR | | logHR OS | -0.01  NR  NR | 0.13  0.09  0.16 |  |  |
| Anti-angio, anti-angiogenic; CR, complete response; diff, difference; HR, hazard ratio; ln, natural logarithm; log, logarithm; NHL, non-Hodgkin’s lymphoma; NR, not reported; NSCLC, non-small cell lung cancer; OR, odds ratio; ORR, overall response rate; OS, overall survival; PFS, progression-free survival; pub, published; recr, recruited; rr, relative risk; SCLC, small cell lung cancer. | | | | | | | | | | | | | |

Supplementary Table 12: Surrogate threshold effect (STE) data reported per study

| **Surrogate relationship** | **Cancer types and refs** | **Based on difference in response** | | | | **Based on odds ratio for response** | | | |  |
| --- | --- | --- | --- | --- | --- | --- | --- | --- | --- | --- |
|  |  | **Surrogate** | **Final** | **STE** | **Surrogate** | | **Final** | **STE** |  |  |
| **ORR to PFS** | Various^77^ | Diff ORR | HR PFS | 15% |  | |  |  |  |  |
| **ORR to OS** | Colorectal^26^ |  |  |  | OR ORR | | OR OS | 0.28 |  |  |
|  | NSCLC^39^ | Diff ORR  Diff ORR | HR OS  Diff median OS | 55%  41% |  | |  |  |  |  |
|  | Various^77^ | Diff ORR | HR OS | 21% |  | |  |  |  |  |
| **CR to PFS** | NHL^69^ |  |  |  | OR CR 30mo | | HR PFS | 1.56 |  |  |
| CR, complete response; diff, difference; HR, hazard ratio; NHL, non-Hodgkin’s lymphoma; NSCLC, non-small cell lung cancer; OR, odds ratio; ORR, overall response rate; OS, overall survival; PFS, progression-free survival; STE, surrogate threshold effect. | | | | | | | | | | |

Supplementary Figure 1: IQWiG scores for strength of association across all 202 analyses (within 63 included studies)


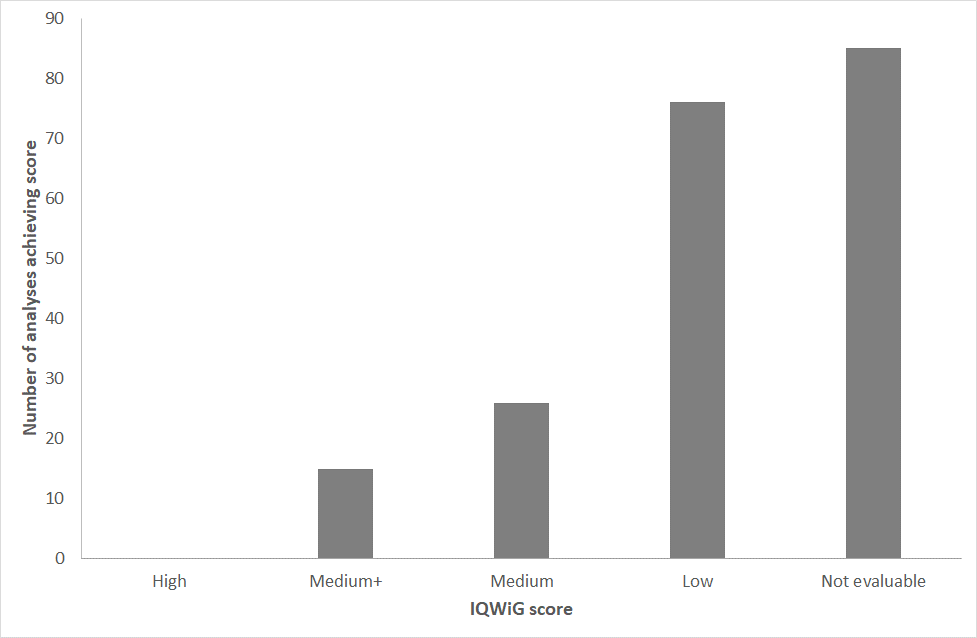


Supplementary Figure 2: BSES2 scores for strength of association across all 202 analyses (within 63 included studies)


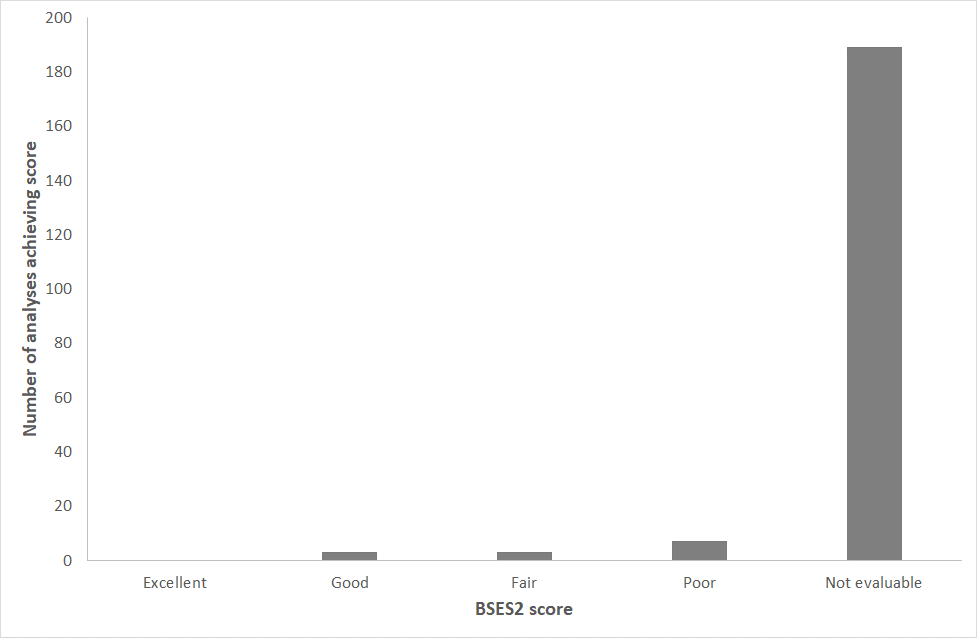

Supplement: Supplementary file 1 — Supplementary Information [file 41416_2020_1050_MOESM1_ESM.docx]
